# The Impact of Model Assumptions on Personalized Lung Cancer Screening Recommendations

**DOI:** 10.1177/0272989X241249182

**Published:** 2024-05-13

**Authors:** Kevin ten Haaf, Koen de Nijs, Giulia Simoni, Andres Alban, Pianpian Cao, Zhuolu Sun, Jean Yong, Jihyoun Jeon, Iakovos Toumazis, Summer S. Han, G. Scott Gazelle, Chung Ying Kong, Sylvia K. Plevritis, Rafael Meza, Harry J. de Koning

**Affiliations:** Department of Public Health, Erasmus MC, University Medical Center Rotterdam, Rotterdam, the Netherlands; Department of Public Health, Erasmus MC, University Medical Center Rotterdam, Rotterdam, the Netherlands; Department of Biomedical Data Sciences, Stanford University, Stanford, CA, USA; MGH Institute for Technology Assessment, Harvard Medical School, Boston, MA, USA; Department of Epidemiology, School of Public Health, University of Michigan, Ann Arbor, MI, USA; Canadian Partnership Against Cancer, Toronto, ON, Canada; Canadian Partnership Against Cancer, Toronto, ON, Canada; Department of Epidemiology, School of Public Health, University of Michigan, Ann Arbor, MI, USA; Department of Health Services Research, The University of Texas MD Anderson Cancer Center, Houston, TX, USA; Quantitative Sciences Unit, Department of Medicine, Stanford University, Stanford, CA, USA; Department of Radiology, Massachusetts General Hospital, Boston, MA, USA; Division of General Internal Medicine, Department of Medicine, Mount Sinai Hospital, New York, NY, USA; Department of Biomedical Data Sciences, Stanford University, Stanford, CA, USA; Department of Integrative Oncology, BC Cancer Research Institute, BC, Canada; School of Population and Public Health, University of British Columbia, BC, Canada; Department of Public Health, Erasmus MC, University Medical Center Rotterdam, Rotterdam, the Netherlands

**Keywords:** lung cancer, personalized screening, natural-history modelling, maximum clinical incidence reduction

## Abstract

**Background:**

Recommendations regarding personalized lung cancer screening are being informed by natural-history modeling. Therefore, understanding how differences in model assumptions affect model-based personalized screening recommendations is essential.

**Design:**

Five Cancer Intervention and Surveillance Modeling Network (CISNET) models were evaluated. Lung cancer incidence, mortality, and stage distributions were compared across 4 theoretical scenarios to assess model assumptions regarding 1) sojourn times, 2) stage-specific sensitivities, and 3) screening-induced lung cancer mortality reductions. Analyses were stratified by sex and smoking behavior.

**Results:**

Most cancers had sojourn times <5 y (model range [MR]; lowest to highest value across models: 83.5%–98.7% of cancers). However, cancer aggressiveness still varied across models, as demonstrated by differences in proportions of cancers with sojourn times <2 y (MR: 42.5%–64.6%) and 2 to 4 y (MR: 28.8%–43.6%). Stage-specific sensitivity varied, particularly for stage I (MR: 31.3%–91.5%). Screening reduced stage IV incidence in most models for 1 y postscreening; increased sensitivity prolonged this period to 2 to 5 y. Screening-induced lung cancer mortality reductions among lung cancers detected at screening ranged widely (MR: 14.6%–48.9%), demonstrating variations in modeled treatment effectiveness of screen-detected cases. All models assumed longer sojourn times and greater screening-induced lung cancer mortality reductions for women. Models assuming differences in cancer epidemiology by smoking behaviors assumed shorter sojourn times and lower screening-induced lung cancer mortality reductions for heavy smokers.

**Conclusions:**

Model-based personalized screening recommendations are primarily driven by assumptions regarding sojourn times (favoring longer intervals for groups more likely to develop less aggressive cancers), sensitivity (higher sensitivities favoring longer intervals), and screening-induced mortality reductions (greater reductions favoring shorter intervals).

**Implications:**

Models suggest longer screening intervals may be feasible and benefits may be greater for women and light smokers.

**Highlights:**

- Natural-history models are increasingly used to inform lung cancer screening, but causes for variations between models are difficult to assess.
- This is the first evaluation of these causes and their impact on personalized screening recommendations through easily interpretable metrics.
- Models vary regarding sojourn times, stage-specific sensitivities, and screening-induced lung cancer mortality reductions.
- Model outcomes were similar in predicting greater screening benefits for women and potentially light smokers. Longer screening intervals may be feasible for women and light smokers.

## Introduction

Lung cancer screening reduces lung cancer mortality by more than 20%.^1,2^ Furthermore, inviting individuals for lung cancer screening based on individual risk has been shown to be (cost-)effective.^3–5^ Findings from trials suggest that the effectiveness and efficiency of lung cancer screening may be improved through personalization.^6–12^

For example, modeling studies have suggested that personalizing the screening stopping age based on life expectancy could considerably reduce overdiagnosis and the number of screens required while retaining the life-years gained by screening.^3,10^ This has led to recommendations to personalize screening by incorporating an individual’s life expectancy.^13,14^

The information provided by the result of the computed tomography (CT) screening may also be used to personalize screening. Analyses of both the National Lung Screening Trial (NLST) and the Dutch–Belgian lung-cancer screening trial (NELSON) indicate that the risk for developing lung cancer was substantially lower among those with a negative baseline screening result compared with those with indeterminate or positive screening results.^6,7^ Consequently, multiple trials are currently evaluating personalizing screening intervals based on the baseline screen result.^15,16^ Furthermore, studies suggest that combining the results of the CT with information from biomarkers may allow even further personalization of the screening interval.^12^

While trials are essential to provide information on the effectiveness of personalized screening regimens, they are restricted to evaluating a single design (e.g., annual versus biennial screening) and population. Consequently, obtaining trial-based evidence for personalized screening regimens based on different combinations of individual characteristics (e.g., individual risk factors, lung cancer risk, expected life expectancy, previous screening results) would be infeasible in practice.

Natural-history models explicitly model the preclinical progression and probability of screening to detect a disease in a preclinical phase. These models are valuable in extrapolating trial results to different program designs and target populations to inform screening recommendations.^17,18^ Furthermore, comparative modeling analyses (evaluations comparing multiple models with standardized inputs) can provide more reliable and robust conclusions than single-model studies.^19^ Consequently, personalized lung cancer screening recommendations are likely to continue to be informed by collaborative modeling analyses.^17^ However, similar to risk-prediction models, equally valid natural-history models can vary in predicted outcomes due to differences in model structures, assumptions, and data used for model calibration.^20–22^ In particular, assumptions regarding

1. screening test sensitivity by stage, affecting the overall potential of the model to detect cancers present in a preclinical state;
2. sojourn time (the preclinical, screen-detectable phase) lengths, affecting the overall duration that a cancer can be detected by screening in the model; and
3. treatment effectiveness for (screen-detected) cancers, affecting the potential benefit a model may attribute to the early detection of a cancer

can vary widely between models.^22–24^ Therefore, it is essential to understand how model structures and assumptions influence personalized lung cancer screening recommendations.

Detailed descriptions and comprehensive documentation contribute to model transparency; however, the implications of differences in model structures and assumptions remain difficult to assess. The maximum clinical incidence reduction (MCLIR) methodology has been specifically designed to compare natural-history assumptions between models.^22^ In brief, the MCLIR methodology evaluates the model-specific reductions in incidence and mortality achievable through screening across different scenarios to evaluate model differences in sojourn times, test sensitivity, and mortality reduction achieved through screening (screening-induced mortality reduction).

The MCLIR methodology has been used to evaluate breast, cervical, and colorectal cancer screening models.^22–24^ In this study, we extend the MCLIR methodology to evaluate how differences in model assumptions regarding test sensitivity, sojourn time lengths, and treatment effectiveness for (screen-detected) cancers affect personalized lung cancer screening recommendations.

## Materials and Methods

This study evaluated 5 Cancer Intervention and Surveillance Modeling (CISNET) models that informed US and Canadian lung cancer screening recommendations.^17,25,26^ These models are the Microsimulation Screening Analysis (MISCAN; Erasmus University Medical Center Rotterdam) model, the Lung Cancer Natural History and Screening (UoM/BCC; University of Michigan/BC Cancer) model, the Lung Cancer Outcomes Simulation (LCOS; Stanford University) model, the Lung Cancer Policy Model (LCPM; Massachusetts General Hospital), and the Oncosim model (Canadian Partnership Against Cancer/Statistics Canada).^27–31^ An overview of the models (Supplementary Table 1) and individual model profiles are provided in the Supplementary Material.

A general model structure overview is provided in Supplementary Figure 1. Each model simulates individuals who may develop lung cancer in the absence/presence of screening. All models simulate lung carcinogenesis through a smoking dose-response model. When lung carcinogenesis occurs, a sojourn time (the preclinical, screen-detectable phase) from tumor onset until clinical diagnosis is simulated. Tumors may undergo preclinical progression to more advanced stages (through stage transition, tumor size increase, or metastatic burden). Upon clinical detection, survival functions (which may vary by tumor and individual characteristics) determine when lung cancer death occurs. Screening may affect a person’s life history through earlier detection of the tumor. The time between screen detection and diagnosis in the absence of screening is known as the lead time. Screening-induced benefits may manifest themselves through improvements in survival due to a stage shift and/or the probability of successful curative treatment.

### MCLIR Methodology

The impact of modeling structures and assumptions is evaluated by comparing lung cancer incidence (clinical and screen detected) and mortality across 4 scenarios. Table 1 provides an overview of the different scenarios, MCLIR metrics, and their implications.

**Table 1:** Overview of the Different Scenarios, Interpretation, and Derivable Maximum Clinical Incidence Reduction (MCLIR) Methodology Metrics

Scenario	Description	Effect of Screening	Interpretation	Derivable MCLIR Metrics
NoScreen	No screening is performed during an individual’s lifetime. Lung cancers that are diagnosed due to clinical symptoms are treated with guideline-concordant treatment with observed treatment effectiveness.	No screening is performed. All cancers in this scenario are diagnosed due to clinical symptoms and are treated with guideline-concordant treatment with observed treatment effectiveness/survival.	The NoScreen Scenario serves as a comparator to evaluate the effects of screening in the other scenarios.	None.
RealSensRealTreat	A single CT screen with realistic sensitivity estimates is performed at age 65 y. All screen-detected cancers receive guideline-concordant treatment with observed treatment effectiveness.	A proportion of the existing cancers at age 65 y will be screen detected. Both screen-detected cancers and cancers diagnosed due to clinical symptoms are treated with guideline-concordant treatment with observed treatment effectiveness/survival.	The comparison of RealSensRealTreat to NoScreen allows derivation of the model predictions for how screening reduces incidence and mortality under its baseline (realistic) assumptions.	Realistic clinical incidence reduction (RCLIR): $\mathrm{RCLIR}=\left(1-\;\frac{\sum_{A=\;\mathrm{age}\;65}^{\mathrm{age}\;80}\mathrm{Clinical}\;\mathrm{incidence}\;\mathrm{at}\;\mathrm{age}\;A\;\mathrm{in}\;\mathrm{RealSensRealTreat}}{\sum_{A=\;\mathrm{age}\;65}^{\mathrm{age}\;80}\mathrm{Clinical}\;\mathrm{incidence}\;\mathrm{at}\;\mathrm{age}\;A\;\mathrm{in}\;\mathrm{NoScreen}}\right)*100\%$ Realistic screening-induced lung cancer mortality reduction (RMOR): $\mathrm{RMOR}=\left(1-\;\frac{\sum_{A=\;\mathrm{age}\;65}^{\mathrm{age}\;80}\mathrm{Lung}\;\mathrm{cancer}\;\mathrm{mortality}\;\mathrm{at}\;\mathrm{age}\;A\;\mathrm{in}\;\mathrm{RealSensRealTreat}}{\sum_{A=\;\mathrm{age}\;65}^{\mathrm{age}\;80}\mathrm{Lung}\;\mathrm{cancer}\;\mathrm{mortality}\;\mathrm{at}\;\mathrm{age}\;A\;\mathrm{in}\;\mathrm{NoScreen}}\right)*100\%$
RealSensPerfectTreat	A single CT screen with realistic sensitivity estimates is performed at age 65 y. All screen-detected cancers receive perfect treatment (i.e., have a 100% lifetime survival rate).	A proportion of the existing cancers at age 65 y will be screen detected. However, all of the screen-detected cancers receive perfect treatment and will not lead to lung cancer death. Lung cancers that develop after age 65 y can be diagnosed only due to clinical symptoms and are treated with guideline-concordant treatment with observed treatment effectiveness/survival.	The comparison of RealSensRealTreat to RealSensPerfectTreat allows derivation of the potential effects of improvements in the treatment of screen-detected cases on lung cancer mortality under baseline sensitivity estimates.	Screening-induced lung cancer mortality reduction under perfect treatment but imperfect sensitivity (ISMOR): $\mathrm{ISMOR}=\left(1-\;\frac{\sum_{A=\;\mathrm{age}\;65}^{\mathrm{age}\;80}\mathrm{Lung}\;\mathrm{cancer}\;\mathrm{mortality}\;\mathrm{at}\;\mathrm{age}\;A\;\mathrm{in}\;\mathrm{RealSensPerfectTreat}}{\sum_{A=\;\mathrm{age}\;65}^{\mathrm{age}\;80}\mathrm{Lung}\;\mathrm{cancer}\;\mathrm{mortality}\;\mathrm{at}\;\mathrm{age}\;A\;\mathrm{in}\;\mathrm{NoScreen}}\right)*100\%$
PerfectSensTreat	A single screen with perfect sensitivity (100%) is performed at age 65 y. All screen-detected cancers receive perfect treatment (i.e., have a 100% lifetime survival rate).	All existing cancers at age 65 are screen-detected. Furthermore, all of the screen-detected cancers receive perfect treatment and will not lead to lung cancer death. Lung cancers that develop after age 65 can only be diagnosed due to clinical symptoms and are treated with guideline-concordant treatment with observed treatment effectiveness/survival.	The comparison of PerfectSensTreat to NoScreen provides the maximum clinical incidence reduction (MCLIR), by removing all existing cancers through screen detection. The MCLIR thus provides an overview of the sojourn time distribution, with the time between the moment of screening and the MCLIR curve reaching 0% reflecting the maximum sojourn time. In addition, the comparison between PerfectSensTreat and NoScreen provides the maximum mortality reduction (MMOR) achievable by screening, as all cancers that exist at the moment of screening are detected and treated with perfect effectiveness. Finally, the comparison between PerfectSensTreat and RealSensPerfectTreat also provides information on the value of improvements in sensitivity on screening-induced lung cancer mortality reductions.	Maximum clinical incidence reduction (MCLIR): $\mathrm{MCLIR}=\left(1-\;\frac{\sum_{A=\;\mathrm{age}\;65}^{\mathrm{age}\;80}\mathrm{Clinical}\;\mathrm{incidence}\;\mathrm{at}\;\mathrm{age}\;A\;\mathrm{in}\;\mathrm{PerfectSensTreat}}{\sum_{A=\;\mathrm{age}\;65}^{\mathrm{age}\;80}\mathrm{Clinical}\;\mathrm{incidence}\;\mathrm{at}\;\mathrm{age}\;A\;\mathrm{in}\;\mathrm{NoScreen}}\right)*100\%$ Screening-induced maximum lung cancer mortality reduction (MMOR): $\mathrm{MMOR}=\left(1-\;\frac{\sum_{A=\;\mathrm{age}\;65}^{\mathrm{age}\;80}\mathrm{Lung}\;\mathrm{cancer}\;\mathrm{mortality}\;\mathrm{at}\;\mathrm{age}\;A\;\mathrm{in}\;\mathrm{PerfectSensTreat}}{\sum_{A=\;\mathrm{age}\;65}^{\mathrm{age}\;80}\mathrm{Lung}\;\mathrm{cancer}\;\mathrm{mortality}\;\mathrm{at}\;\mathrm{age}\;A\;\mathrm{in}\;\mathrm{NoScreen}}\right)*100\%$

CT, computed tomography.

In brief, a scenario without screening (NoScreen) is compared with 3 scenarios evaluating a 1-time screen at age 65 y (the center of the US Preventive Services Task Force–recommended screening age range) under different assumptions.

Scenario RealSensRealTreat evaluates screening with realistic sensitivity and treatment effectiveness estimates. Thus, in this scenario, the models apply their regular stage-specific screening sensitivity and effect of screening on influencing lung cancer mortality. The comparison to NoScreen thus provides information on the model’s realistic clinical incidence reduction (RCLIR) and realistic screening-induced lung cancer mortality reduction (RMOR). These metrics reflect model predictions under their baseline assumptions.

Scenario RealSensPerfectTreat evaluates a screening test with realistic sensitivity estimates and perfect treatment of all screen-detected cancers. This is facilitated in the models through curing all screen-detected cancers or setting their survival to 100%. While RealSensPerfectTreat and RealSensRealTreat screen detect the same cancers, those in RealSensPerfectTreat are treated with perfect effectiveness. The difference in mortality reduction between RealSensPerfectTreat and RealSensRealTreat thus represents cancers that were screen detected but still lead to lung cancer death due to imperfect treatment. Consequently, this comparison yields information on the model’s screening-induced lung cancer mortality reduction under perfect treatment but imperfect sensitivity (ISMOR). This metric represents the potential mortality reduction that can be achieved by improvements in treatment effectiveness under current screening sensitivity.

Finally, scenario PerfectSensTreat evaluates a perfect screening test with perfect treatment of all screen-detected cancers. This is facilitated in the models by setting the sensitivity of the screening test to 100% for all stages of cancer. Thus, in this scenario, all cancers present at the time of screening are detected. Consequently, this scenario yields information on the model’s MCLIR. This metric represents the potential incidence reduction achievable through sensitivity improvements. Furthermore, all screen-detected cancers in this scenario receive perfect treatment, yielding information on the model’s screening-induced maximum lung cancer mortality reduction (MMOR). This metric represents the maximum mortality reduction achievable by screening. Finally, the sojourn time distribution can be derived through comparing differences in time-specific clinical incidence between NoScreen and PerfectSensTreat.

The MCLIR metrics (RCLIR/MCLIR/RCLIR; RMOR/ISMOR/MMOR) are derived by comparing the incidence and mortality between scenarios over ages 65 to 80 y, for example, the MCLIR is derived as

$$\mathrm{MCLIR}=\left(1-\frac{\sum_{A=\mathrm{age}\;65}^{\mathrm{age}\;80}\mathrm{Clinical}\;\mathrm{incidence}\;\mathrm{at}\;\mathrm{age}\;A\;\mathrm{in}\;\mathrm{PerfectSensTreat}}{\sum_{A=\;\mathrm{age}\;65}^{\mathrm{age}\;80}\mathrm{Clinical}\;\mathrm{incidence}\;\mathrm{at}\;\mathrm{age}\;A\;\mathrm{in}\;\mathrm{NoScreen}}\right)*100\%.$$

Derivations for all MCLIR metrics are provided in Table 1.

This study expands the MCLIR methodology by evaluating the stage distributions of screen-detected and clinically detected cancers. Comparisons of the ratios of screen-detected cancers by stage between scenarios with perfect (PerfectSensTreat) and realistic (RealSensPerfectTreat/RealSensRealTreat) sensitivity provide insights into the model-specific preclinical prevalence and screen detectability by stage.

Screening aims to diagnose cancers at an early stage: thus, the screening interval length should allow detection before progression to advance disease occurs. Screen detection of early-stage cancers before they progress to advanced disease will reduce the clinical incidence of late-stage disease. Therefore, we evaluated the incidence of clinically detected stage IV cancers postscreening to attain insights into model-specific optimal screening intervals.

### Evaluated Populations

Screening-induced lung cancer mortality reductions may vary by sex.^1,32^ Furthermore, individuals with high lung cancer risk are more likely to have comorbidities that may affect treatment options.^33,34^ The scenarios were evaluated by sex and for different smoking behaviors to provide insights into screening-effectiveness differences across these characteristics. Two smoking behaviors were considered. The first was heavy smoking, defined as starting smoking at age 15 y, never quitting, and smoking 30 cigarettes per day over the smoking lifetime (i.e., accumulating 75 pack-years and currently smoking at age 65 y). The second was light smoking, defined as starting smoking at age 15 y, quitting at age 55 y, and smoking 10 cigarettes per day over the smoking lifetime (i.e., accumulating 20 pack-years and having quit 10 y at age 65 y). The 6 populations considered were thus 1) heavy smokers (both sexes combined), 2) light smokers (both sexes combined), 3) heavy-smoking men, 4) light-smoking men, 5) heavy-smoking women, and 6) light-smoking women. In each analysis, smoking behaviors were uniform across the simulated population to allow standardization across models.

### Outcome Definitions

Heavy smokers (both sexes combined) were considered for the primary analyses; the results for the other populations are provided in the supplementary materials. All individuals were assumed to never have received prior screening or been diagnosed with lung cancer before age 65 y. All individuals were assumed to die of other causes at age 100 y to account for differences in smoking-related mortality across models. All outcome metrics were calculated by comparing differences in outcomes between the NoScreen, RealSensRealTreat, RealSensPerfectTreat, and PerfectSensTreat scenarios. The outcome metrics are provided as either medians across the models or by range across the models. For example, the model range (MR) represents the range from the lowest to highest value across the models for the specified outcome metric.

## Results

### Model Differences in Stage-Specific Sensitivity

**Figure 1:**
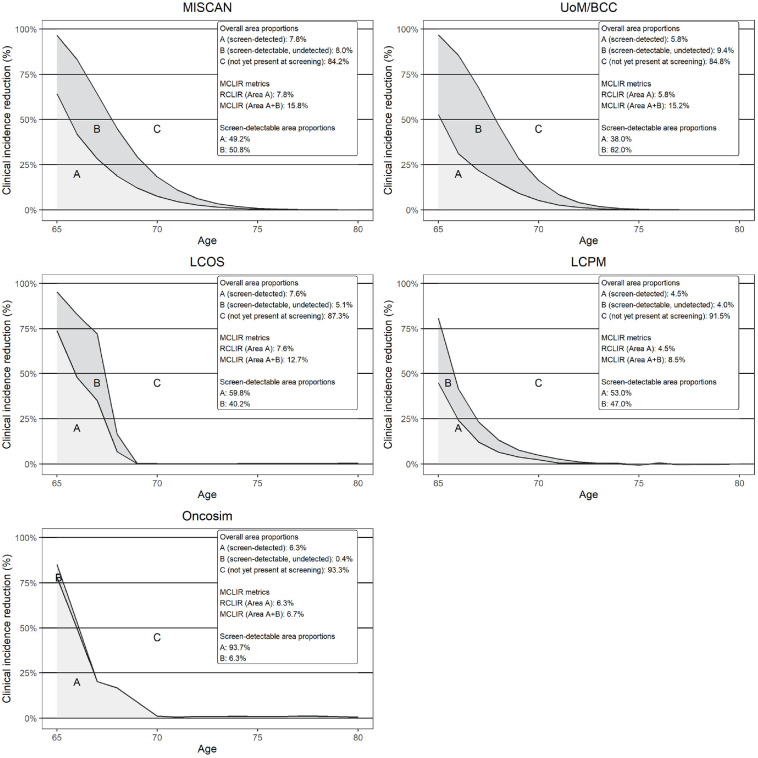
Fifteen-year model-specific lung cancer incidence reductions under different assumptions. The *y*-axis refers to the observed reduction in the incidence of clinically detected lung cancers compared with no screening at each age across the different modeled scenarios. The top part of the legend (overall area proportions) refers to the proportion of lung cancers occurring over ages 65 to 80 y in each of the areas A, B, and C, which represent cancers occurring over ages 65 to 80 y. Area A is derived by comparing the cancer incidence in scenarios RealSensPerfectTreat /RealSensRealTreat to scenario NoScreen and forms the RCLIR. It shows the proportion of cancers occurring over ages 65 to 80 y that are detected when assuming realistic sensitivity estimates. Area B is derived by comparing the cancer incidence in scenario PerfectSensTreat to scenarios RealSensPerfectTreat/RealSensRealTreat to NoScreen. It shows the proportion of cancers that are present at the moment of screening but are not detected due to imperfect sensitivity. Areas A and B combined form the MCLIR. Finally, area C represents the proportion of cancers that will be detected clinically between ages 65 to 80 y but that are not yet present at the moment of screening. For example, in MISCAN, 7.8% (area A) of cancers occurring over ages 65 to 80 y are detected at the moment of screening, while 8.0% (area B) is missed due to imperfect sensitivity and 84.2% (area C) is not yet detectable at the moment of screening. Consequently, MISCAN’s RCLIR is 7.8% and its MCLIR 15.8%. Of the cancers that are detectable at the moment of screening, MISCAN detects 49.2% (area A) but misses 50.8% (area B) due to imperfect sensitivity. The bottom part of the legend refers to the distribution of areas A and B when the cancers detectable at the moment of screening are considered as the denominator (areas A+B).

Differences in RCLIRs (Figure 1, area A) were modest (MR: 4.5%–7.8%). However, RCLIRs in the year of screening varied widely (MR: 44.7%–78.0%). This suggests screening sensitivity variability across models, as shown in Table 2. Stage I sensitivity varied considerably, ranging from 31.3% to 91.5% (median: 43.5%). This reflects differences in model structures as the upper bound was derived from a model that assumes a similar sensitivity across all stages. Similarly, sensitivity also varied for more advanced cancers, MRs 35.1% to 90.8% (median: 61.8%) for stage II, 62.7% to 92.2% (median: 90.2%) for stage III, and 86.4% to 98.0% (median: 91.4%) for stage IV. Overall, models with low sensitivities for early-stage cancers were more likely to favor shorter screening intervals, as they required more opportunities to detect similar numbers of early-stage cancers as models with high sensitivities.

**Table 2:** Screening Sensitivity Differences between Models^a^

Model	Stage I Sensitivity	Stage II Sensitivity	Stage III Sensitivity	Stage IV Sensitivity
MISCAN	40.3%	43.9%	71.3%	98.0%
UoM/BCC	31.3%	35.1%	62.7%	87.1%
LCOS	52.1%	61.8%	92.2%	92.6%
LCPM	43.5%	67.0%	90.2%	86.4%
Oncosim	91.5%	90.8%	91.2%	91.4%
Median	43.5%	61.8%	90.2%	91.4%

LCOS, Lung Cancer Outcomes Simulation model; LCPM, Lung Cancer Policy Model; MISCAN, Microsimulation Screening Analysis model; UoM/BCC, Lung Cancer Natural History and Screening model.

a At the time of the analysis, Oncosim’s assumed screening sensitivity depends on screening round and age group and did not explicitly model stage-specific sensitivities. Rather, it assigns a stage to a clinically detected cancer consistent with national cancer registry data (by sex, age group, and jurisdiction) and a stage to a screen-detected cancer upon the moment of detection that is consistent with the National Lung Screening Trial results.

### Model Differences in Sojourn Times

All models assume that most cancers between ages 65 to 80 y develop postscreening, demonstrated by the MCLIRs (Figure 1, areas A + B) ranging from 6.7% to 15.8% (median: 12.7%). Indeed, all models assumed most cancers had sojourn times <5 y (MR: 83.5%–98.7% of cancers). However, cancer aggressiveness still varied across models, demonstrated by differences in proportions of cancers with sojourn times <2 y (MR: 42.5%–64.6%) and 2 to 4 y (MR: 28.8%–43.6%). This is also reflected in the MCLIR shapes. For example, while all models show steep declines in MCLIR in the first 3 y postscreening, the LCOS and Oncosim MCLIRs further decline to nearly 0% in the subsequent 3 y. The other MCLIRs show slower declines toward 0%, resulting in longer tails. Both patterns reflect mixtures of highly aggressive cancers with short sojourn times and less aggressive cancers with longer sojourn times. However, the differences in the rates of decline suggest that the sojourn times of less aggressive cancers are generally longer in the models with longer MCLIR tails. Consequently, if individuals who develop less aggressive cancers can be identified, models with longer sojourn times are more likely to recommend longer screening intervals.

### Effects of Sensitivity and Sojourn Time Assumptions on the Screening Interval

The stage distributions in the absence of screening (Supplementary Figure 2) and of screen-detected cases under perfect sensitivity (Supplementary Figure 3) are similar across models. Thus, preclinical stage distributions and maximum potential stage shifts at the first screening are similar across models. However, at similar screening intervals, models with shorter sojourn times may have more interval cancers than those with longer sojourn times and may be less likely to recommend longer intervals.

**Figure 2:**
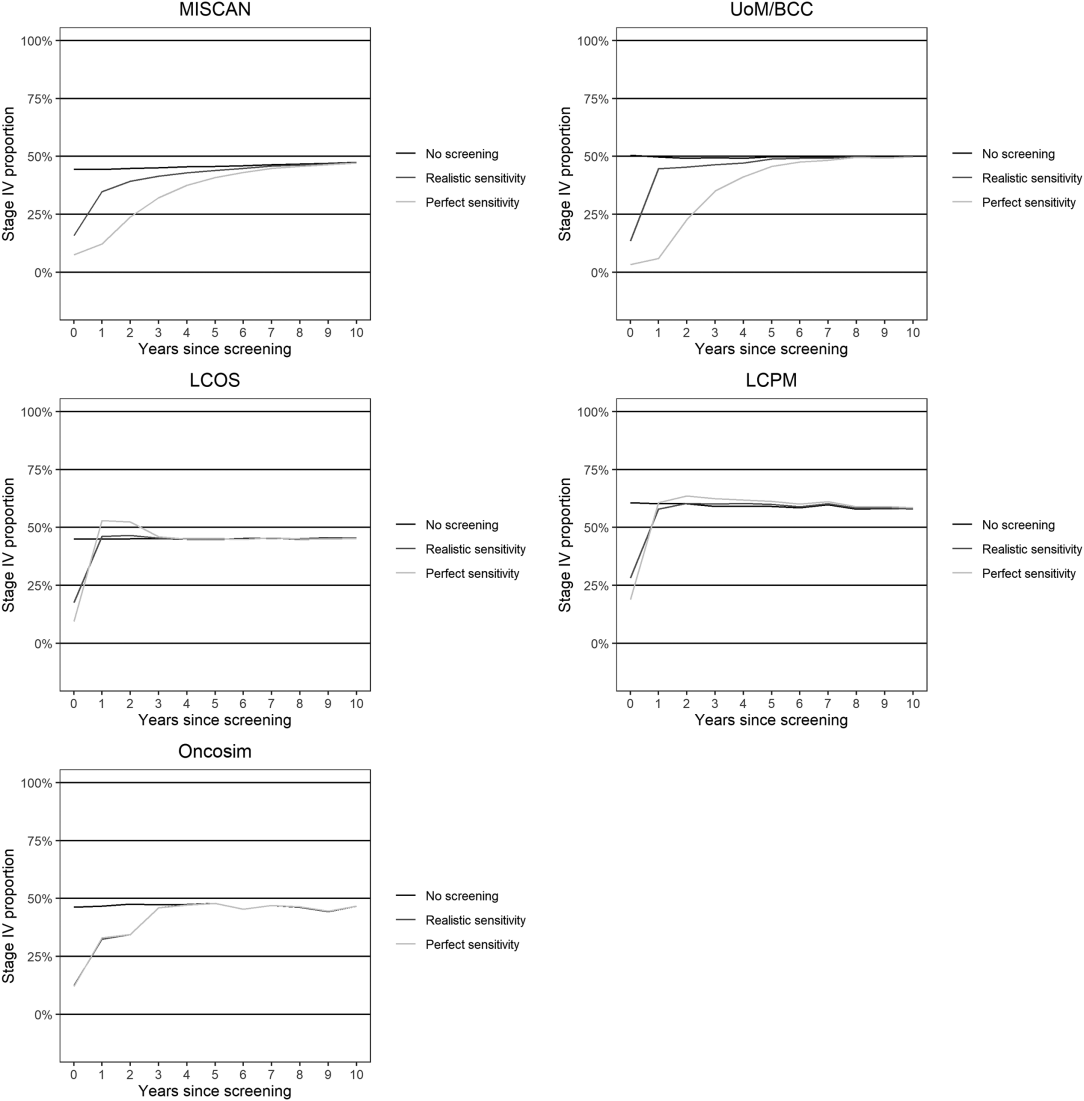
Effect of sensitivity on the occurrence of stage IV cancers postscreening. The figure notes the proportion of clinically detected lung cancers occurring in stage IV (on the *y*-axis) in each year postscreening under the different scenarios. The line “No screening” represents the proportion of stage IV cancers in scenario NoScreen. The line “Realistic sensitivity” represents the proportion of stage IV cancers in scenarios RealSensPerfectTreat and RealSensRealTreat. Finally, the line “Perfect sensitivity” represents the proportion of stage IV cancers in scenario PerfectSensTreat.

The relation between sensitivity and the screening interval is also demonstrated in Figure 2, which shows how sensitivity affects the postscreening incidence of stage IV cancers. Screening causes a postscreening reduction in stage IV cancers in most models. This effect is short lived and declines substantially after 1 y postscreening under realistic sensitivity. However, when perfect sensitivity is assumed, the reduction in stage IV cancers prolongs to 2 to 5 y postscreening. Overall, these results indicate that models with higher sensitivities are more likely to favor longer screening intervals.

### Screening-Induced Lung Cancer Mortality Reductions

**Figure 3:**
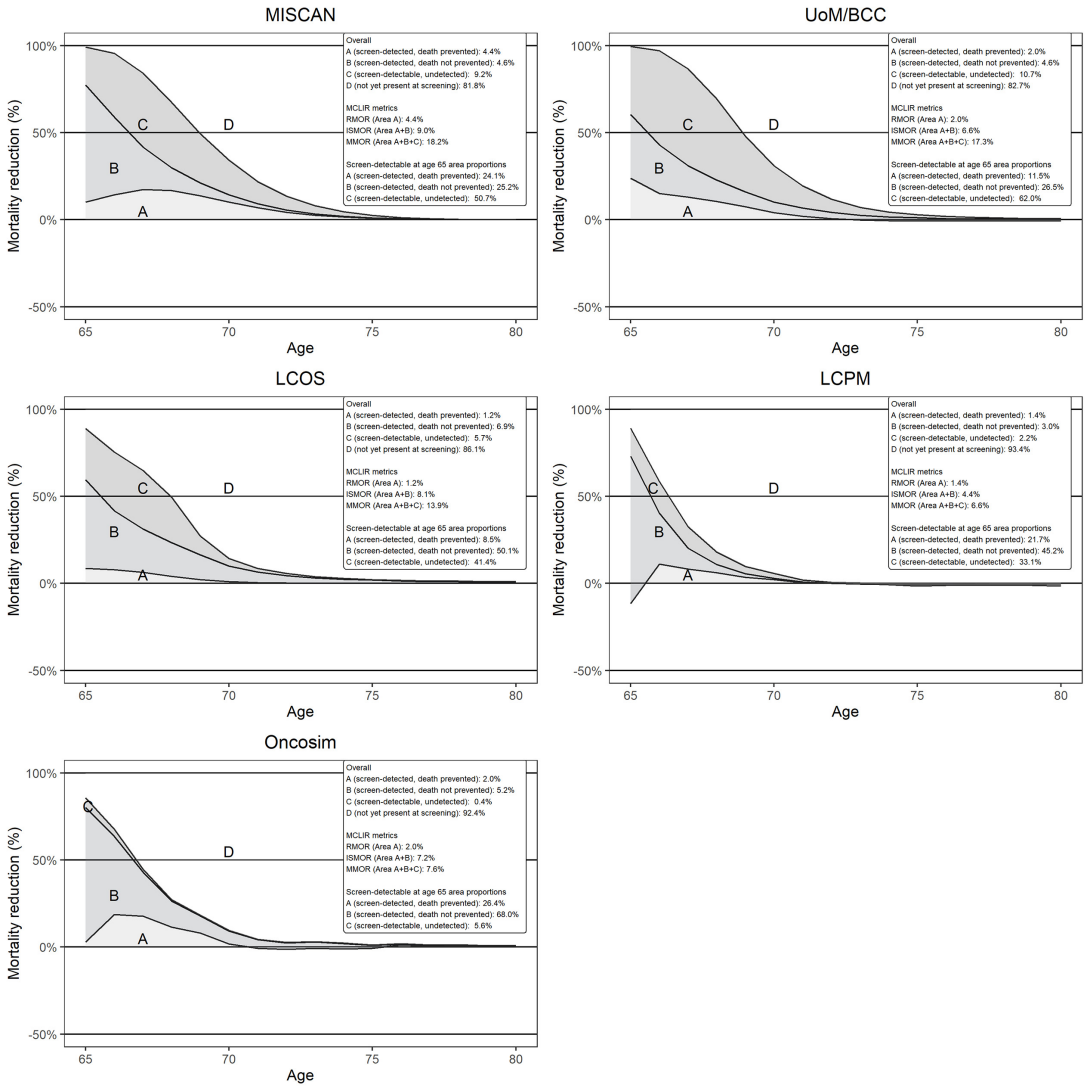
Five-year model-specific lung cancer mortality reductions under different assumptions. The *y*-axis of the graphs refers to the observed reductions in lung cancer mortality on the population level compared with no screening at each age across the different modeled scenarios. The top part of the legend refers to the proportion of lung cancer deaths occurring under no screening over ages 65 to 80 y in each of the areas A, B, C, and D. Area A is derived by comparing the cancer mortality in scenario RealSensRealTreat to scenario NoScreen and forms the RMOR. It represents the proportion of cancer deaths occurring over ages 65 to 80 y that are detected when assuming realistic sensitivity estimates and for which death is prevented when realistic treatment is assumed. Area B is derived by comparing the cancer mortality in scenario RealSensPerfectTreat to scenario RealSensRealTreat. It represents the proportion of cancers leading to death that are detected at the moment of screening under realistic sensitivity assumptions but for which death is not prevented due to imperfect treatment. Areas A and B combined form the ISMOR. Area C is derived by comparing the cancer mortality in scenario PerfectSensTreat to scenario RealSensPerfectTreat. It represents the proportion of deaths due to cancers that are present at the moment of screening but that are not detected due to imperfect sensitivity. Areas A, B, and C combined form the MMOR. For example, in UoM/BCC, 2.0% (area A) of cancers leading to deaths occurring over ages 65 to 80 y are detected at the moment of screening and successfully treated when realistic assumptions are used. Meanwhile, 4.6% (area B) is detected but unsuccessfully treated; 10.7% (area C) is missed due to imperfect sensitivity and 82.7% (area D) of deaths are due to cancers not yet detectable at the moment of screening. Consequently, UoM/BCCs RMOR is 2.0%, its ISMOR is 6.6%, and its MMOR is 17.3%. Of the deaths due to cancers that are detectable at the moment of screening, UoM/BCC detects and successfully treats 11.5% (area A), detects but unsuccessfully treats 26.5% (area B) and misses 62.0% (area C) due to imperfect sensitivity. The bottom part of the legend refers to the distribution of areas A, B, and C when deaths due to cancers that are detectable at the moment of screening are considered as the denominator.

Figure 3 shows the model-specific lung cancer mortality reductions under different assumptions. The RMORs (Figure 3, area A) ranged from 1.2% to 4.4% (median: 2.0%) while the ISMORs (Figure 3, areas A + B) ranged from 6.6% to 9.0% (median: 7.2%). The within-model difference in ISMOR and RMOR reflects deaths not prevented due to imperfect treatment. This difference was the greatest for LCOS (RMOR: 1.2%; ISMOR: 8.1%) and smallest for MISCAN (RMOR: 4.4%; ISMOR: 9.0%), reflecting differences in treatment effectiveness assumptions.

The MMORs (Figure 3, areas A+B+C) ranged from 6.6% to 18.2% (median: 13.9%), which, similarly to the MCLIRs, reflect that most deaths occur from lung cancers that developed postscreening. The within-model difference in MMOR and ISMOR reflects the cancer deaths not prevented due to imperfect sensitivity. This difference was greatest for UoM/BCC (ISMOR: 6.6%; MMOR: 17.3%) and smallest for Oncosim (ISMOR: 7.2%; MMOR: 7.6%), reflecting differences in sensitivity assumptions (Table 1).

### Effects of Screening-Induced Mortality Reduction Assumptions on the Estimated Value of Improved Sensitivity and the Optimal Screening Interval

Table 3 further demonstrates differences in screening-induced lung cancer mortality reductions. The proportion of screen-detectable lung cancers resulting in mortality missed by screening varied from 5.6% to 62.0%, reflecting great differences in the potential benefits of sensitivity improvements across models. Furthermore, the proportion of screen-detected cases for which mortality was prevented varied from 14.6% to 48.9%, indicating vast differences in the estimated treatment effectiveness of screen-detected cases. For example, while screen detection in LCOS reduced lung cancer mortality by 14.6%, this was 30.2% in UoM/BCC. Consequently, equal improvements in sensitivity will more likely lead to notable improvements in lung cancer mortality reductions for UoM/BCC than LCOS. Furthermore, models with greater treatment effectiveness of screen-detected cases are more likely to recommend shorter intervals that maximize the number of opportunities to detect curable cancer.

**Table 3:** Screening Effectiveness in Reducing Lung Cancer Mortality

Model	Proportions of All Cancers Leading to Lung Cancer Death Detectable at Screening			Proportions of Screen-Detected Cases	
	Area A (Death Prevented)	Area B (Detected but Not Prevented)	Area C (Not Detected, not Prevented)	A/A+B (Detected and Prevented)	B/A+B (Detected but Not Prevented)
MISCAN	24.1%	25.2%	50.7%	48.9%	51.1%
UoM/BCC	11.5%	26.5%	62.0%	30.2%	69.8%
LCOS	8.5%	50.1%	41.4%	14.6%	85.4%
LCPM	21.7%	45.2%	33.1%	32.5%	67.5%
Oncosim	26.4%	68.0%	5.6%	28.0%	72.0%
Median	21.7%	45.2%	41.4%	30.2%	69.8%

MISCAN, Microsimulation Screening Analysis model; UoM/BCC, Lung Cancer Natural History and Screening model; LCOS, Lung Cancer Outcomes Simulation model; LCPM, Lung Cancer Policy model. Areas A, B, and C refer to the areas depicted in Figure 3. Area A represents the proportion of cancers leading to death occurring over ages 65 to 80 y that are detected when assuming realistic sensitivity estimates. Area B represents the proportion of cancers leading to death that are detected at the moment of screening but whose death is not prevented due to imperfect treatment. Finally, area C represents the proportion of cancers leading to death that are not detected due to imperfect sensitivity.

### Sex Differences

All models suggest longer sojourn times for women than men (Supplementary Figures 4 and 5). Furthermore, women have more favorable stage distributions in the absence of screening (Supplementary Figures 6 and 7) and for screen-detected cases under perfect sensitivity (Supplementary Figures 8 and 9). Consequently, although clinical diagnosis occurs more often at an early stage for women, the maximum screening-induced stage shift is more favorable than for men. Furthermore, all models showed greater screening sensitivity for women (Supplementary Tables 2 and 3). Due to the longer sojourn times and greater sensitivities, the postscreening reduction in stage IV cancers persists longer in women than in men (Supplementary Figures 10 and 11). Finally, RMORs/ISMORs/MMORs were higher for women than men (Supplementary Figures 12 and 13), suggesting greater screening-induced lung cancer mortality reductions, as shown in Supplementary Tables 4 and 5. Consequently, all models are more likely to predict greater screening-induced lung cancer mortality reductions and recommend longer screening intervals for women than men.

### Smoking Behavior Differences

Most models did not assume sojourn times varied by smoking behavior (Supplementary Figure 14). However in UoM/BCC and LCPM, the histology distribution varies by smoking behavior, leading to a higher proportion of adenocarcinomas in light versus heavy smokers. Consequently, their MCLIRs for light smokers have longer tails compared with heavy smokers, suggesting longer sojourn times. Furthermore, both models estimated more favorable stage distributions in the absence of screening (Supplementary Figure 15) and for screen-detected cases under perfect sensitivity (Supplementary Figure 16) for light smokers. However, only UoM/BCC estimated higher sensitivities for light smokers (Supplementary Table 6). Consequently, UoM/BCC was the only model to estimate longer reductions in postscreening stage IV cancers for light smokers (Supplementary Figure 17). The RMORs/ISMORs/MMORs were higher for light smokers than for heavy smokers (Supplementary Figure 18) in UoM/BCC and LCPM, suggesting greater screening-induced lung cancer mortality reductions (Supplementary Table 7). In contrast, screening effectiveness was greater for heavy smokers in Oncosim.

## Discussion

This is the first study that evaluates how differences in model structures and assumptions affect model-based personalized lung cancer screening recommendations. Our study compared 5 established natural-history models and found that the models had similar stage-shift potentials. However, models varied in assumptions regarding the sojourn times of less aggressive cancers, stage-specific sensitivities, and screening-induced lung cancer mortality reductions. This is particularly important as our study suggests that these assumptions drive model-based personalized screening recommendations.

Sensitivity estimates for stage I cancers ranged from 31.3% to 91.5%. Overall, lower sensitivities are associated with shorter screening intervals, demonstrated by the postscreening stage IV cancer incidence reduction. The median 1-y postscreening proportion of clinically detected stage IV cancers at realistic sensitivity observed across the models was 15.8%, which is in line with that observed after the first screen in the NLST (16.7%).^2^ Our results suggest that improved sensitivity could prolong this period to 2 to 5 y. However, further evaluation of empirical data is required to assess the proportions of interval cancers that represent cancers missed at the CT screening and interval cancers that develop after the screening.

Differences in sensitivity may also affect the estimated value of personalized information. For example, current nodule management protocols suggest including prior screening information (like nodule volume doubling times) or blood-based biomarkers to improve sensitivity at repeat screenings.^9,12,35–38^ However, the additional value of sensitivity improvements depends on the baseline level (e.g., a 10-percentage-point increase is of greater value at a baseline of 30% compared with 80%).

Sensitivity also varied for advanced cancers: even for stage IV lung cancer, the stage-specific sensitivity varied from 86.4% to 98%. Although a sensitivity of <90% for metastatic cancer may seem low, a CT scan may miss cancers that present as small pulmonary nodules but have metastasis in areas not visible on the CT screen of the chest. In the NLST, 14.7% of individuals with stage IV cancers that were either detected at or clinically diagnosed within the year of each screening round had a negative screening result.^2^ Consequently, we believe that the range of estimates for stage IV sensitivities across the models is credible.

Although the difference in prognosis between stages III and IV is more modest than between stages I and IV, differences in sensitivity for advanced cancers may still affect treatment patterns, treatment durations (due to lead time), and associated costs.^39,40^ Consequently, this may affect cost-effectiveness estimates when the costs and effects of novel therapies are considered.^41^ This is especially important considering the wide range in screening-induced mortality reductions (14.6%–48.9%). Similarly to sensitivity, models with low baseline estimates for treatment effectiveness are more likely to attribute greater value to improvements in treatment effectiveness than models with high baseline estimates. Furthermore, models with greater potential for screening-induced mortality reductions are more likely to recommend shorter intervals, maximizing the number of opportunities to screen-detect curable cancer. In addition, an evaluation of the impact of model differences in assumptions on overall survival improvements in the absence of screening may be useful in informing the joint effect of screening and improvements of novel therapies.^41^

More than 80% of cancers in all models had sojourn times <5 y. However, the models varied in the proportions of cancers with sojourn times <2 y (MR: 42.5%–64.6%) and 2 to 4 y (MR: 28.8%–43.6%). Although annual screening intervals have been recommended in the United States, biennial screening intervals may be favored in other countries due to budgetary or CT capacity constraints.^42^ For example, the United Kingdom, Australia, and Switzerland are recommending or using biennial screening intervals.^42–45^ Therefore, personalized screening recommendations in countries using biennial screening may entail shortening the screening interval to annual screening for high-risk groups based on their CT scan result. Consequently, this is being evaluated by screening trials in Europe.^15,16^

All models found longer sojourn times and greater screening-induced lung cancer mortality reductions for women than for men. This is consistent with reported differences in the incidence of less aggressive histological subtypes, such as adenocarcinoma and screening-induced lung cancer mortality reductions between sexes.^1,32,46^ Consequently, all models are more likely to estimate more favorable screening outcomes and suggest longer screening intervals for women. In contrast, only some models considered differences in sojourn times and screening-induced lung cancer mortality reductions by smoking behavior. Therefore, personalized screening recommendations by smoking behavior may vary across the evaluated models.

Lung cancer screening is becoming increasingly personalized.^47^ As further personalization will add increasing complexity to, and increase the potential value of, collaborative modeling analyses, improving model transparency through easily interpretable metrics is vital. Our study serves as a basis for comparing models in a transparent and easily interpretable manner that can be further expanded to different components of the screening pathway. Previous comparative analyses showed that although there is considerable heterogeneity in model assumptions, the models were consistent in supporting particular policies.^17,18,48^ The comparative analyses in this investigation demonstrate that there could be more heterogeneity in models’ assessment of the performance of personalized screening strategies and their (cost-)effectiveness. Consequently, this study demonstrates the value of the MCLIR methodology in identifying areas of heterogeneity between models and assessing how these might influence the estimated impact of different aspects of personalized screening and which strategies are supported. Furthermore, it highlights the need to further assess the validity of model assumptions on data from ongoing trials on personalized lung cancer screening.

Nonetheless, our analysis has some limitations. While between-model differences in sex and smoking behavior were evaluated, the consideration of a single screen at age 65 y precluded evaluating differences in age-specific screening effectiveness and assumptions on information from prior screenings. A previous collaborative analysis suggested that age-specific overdiagnosis risks varied substantially across models.^49^ Screening effectiveness assumptions may similarly vary by age across models. Therefore, future studies should evaluate the MCLIR metrics at different ages and investigate scenarios that consider multiple screenings.

## Conclusion

Our study demonstrates how differences in model assumptions and structures can affect model-based personalized screening recommendations. Differences in model-based personalized screening recommendations are primarily driven by assumptions regarding the sojourn times of less aggressive cancers, screening sensitivity, and screening-induced mortality reductions. Therefore, comparative modeling studies informed by detailed assessment of the impact of model assumptions are vital to inform personalized screening recommendations. Model validation to data from ongoing trials on personalized lung cancer screening is essential to assess the validity of model assessments of personalized screening strategies.

## Supplemental Material

sj-docx-1-mdm-10.1177_0272989X241249182 – Supplemental material for The Impact of Model Assumptions on Personalized Lung Cancer Screening Recommendations Supplemental material, sj-docx-1-mdm-10.1177_0272989X241249182 for The Impact of Model Assumptions on Personalized Lung Cancer Screening Recommendations by Kevin ten Haaf, Koen de Nijs, Giulia Simoni, Andres Alban, Pianpian Cao, Zhuolu Sun, Jean Yong, Jihyoun Jeon, Iakovos Toumazis, Summer S. Han, G. Scott Gazelle, Chung Ying Kong, Sylvia K. Plevritis, Rafael Meza and Harry J. de Koning in Medical Decision Making

sj-docx-2-mdm-10.1177_0272989X241249182 – Supplemental material for The Impact of Model Assumptions on Personalized Lung Cancer Screening Recommendations Supplemental material, sj-docx-2-mdm-10.1177_0272989X241249182 for The Impact of Model Assumptions on Personalized Lung Cancer Screening Recommendations by Kevin ten Haaf, Koen de Nijs, Giulia Simoni, Andres Alban, Pianpian Cao, Zhuolu Sun, Jean Yong, Jihyoun Jeon, Iakovos Toumazis, Summer S. Han, G. Scott Gazelle, Chung Ying Kong, Sylvia K. Plevritis, Rafael Meza and Harry J. de Koning in Medical Decision Making
