# Supplementary material for: The Impact of Model Assumptions on Personalized Lung Cancer Screening Recommendations
Source: Med Decis Making. 2024 May 13;44(5):497–511. doi: 10.1177/0272989X241249182 (PMC11281869; doi:10.1177/0272989X241249182)
Supplement: sj-docx-1-mdm-10.1177_0272989X241249182 – Supplemental material for The Impact of Model Assumptions on Personalized Lung Cancer Screening Recommendations [file sj-docx-1-mdm-10.1177_0272989X241249182.docx]

**Model profiles**

**Microsimulation Screening Analysis (MISCAN) model**

The Microsimulation Screening Analysis (MISCAN) model is a microsimulation model that simulates a population of individual life histories. For each individual, a smoking history (including never smoking) is generated using the Smoking History Generator (SHG) [1]. Lung cancer is modeled through a multistep procedure. Once a person’s age at death from causes other than lung cancer is generated by the SHG, which is influenced by the person’s smoking history, the integrated Two-Stage Clonal Expansion (TSCE) model is used to determine whether lung cancer develops in that individual [2, 3]. MISCAN distinguishes four histological types of lung cancer: squamous cell carcinoma, adenocarcinoma, other non-small cell carcinoma, and small-cell carcinoma.

Once lung cancer has developed, it progresses to more advanced preclinical stages until it is detected. Lung cancers can be detected either clinically (due to symptoms) or by screening. The incidence of clinically detected lung cancers depends on the preclinical duration and the probability of clinical detection, which both vary by cancer stage and histology (and sex for the preclinical duration). Data from the National Lung Screening Trial (NLST) and the Prostate, Lung, Colorectal, and Ovarian Cancer Screening Trial (PLCO) were used to calibrate MISCAN, from which information on the natural history and screen detectability of lung cancer was derived [4-7]. Through incorporating information on the preclinical duration, probability of clinical detection, and screen detectability of lung cancer, as well as a person’s death from causes other than lung cancer, MISCAN can account for the effects of lead time and overdiagnosis.

If screening does not occur, lung cancers can only be clinically detected. If persons die of lung cancer before dying from other causes, their ages of death are adjusted accordingly. Upon activating the screening component, preclinical lung cancers may be detected by screening, which may cure patients, allowing them to resume their normal (cancer-free) life history. The probability of cure differs by the stage of cancer at detection and was based on data from the NLST. Upon clinical or screen detection (if no curation occurs) of lung cancer, the patient is assigned a histological type-, stage- and sex-specific survival time, which follows a piecewise uniform distribution. The model can evaluate various simulated events in the presence and absence of screening, such as the mortality due to lung cancer and other causes, life-years gained, and overdiagnosis.

MISCAN was used for the decision analyses used to inform the U.S. Preventive Services Task Force (USPSTF) for their 2013 and 2021 recommendations for lung cancer screening [8, 9]. The model was used to evaluate the cost-effectiveness of lung cancer screening in Australia, Switzerland and Ontario [10-12]. The model was also used in recent comparative modeling studies by the CISNET Lung Group: 1) evaluation of the effects of risk-stratified screening and 2) analyses of the cost-effectiveness of lung cancer screening in the United States [13-16].

**Lung Cancer Natural History and Screening (UoM/BCC) model**

The Lung Cancer Natural History and Screening (UoM/BCC) model is a combination of a multistage carcinogenesis model and a discrete-state microsimulation model. The model was developed to evaluate the effect of screening on lung cancer incidence and mortality, survival outcomes, overdiagnosis, and quality of life. This model consists of two components: natural history and screening. It was used for the 2013 and 2021 USPSTF lung cancer screening decision analyses [8, 9]. The model was recently used to assess the cost-effectiveness of LDCT in the United States and the effect of risk-based screening strategies in collaboration with the other CISNET Lung groups [13-16].

The natural history component simulates individual lung cancer–oriented life events in the absence of screening given the individual’s smoking history. The age at clinical detection of lung cancer (dose-response) is simulated through a TSCE model [2, 3]. The TSCE model assumes that two mutation (rate-limiting) events are required for the initiation of premalignant lung tumors, and it explicitly models the dynamics of premalignant and malignant tumors. The TSCE model was fitted to lung cancer incidence in the NHS and the HPFS using a likelihood-based approach. If an individual develops clinically diagnosed lung cancer, the natural history model also simulates the age at lung cancer onset, histology, stage at diagnosis, preclinical sojourn time for each stage, and age at lung cancer death. Lung cancer histology is classified into four main groups: small cell, adenocarcinoma, squamous, and other. Histology is simulated using a multinomial logistic regression accounting for sex, age, and smoking exposure model based on the PLCO. Preclinical sojourn times for each stage follow a Weibull distribution with shape and scale parameters depending on sex, stage, and histology [5]. The preclinical sojourn times are simulated for all stages for each clinically diagnosed lung cancer case and used in the screening component to model the effect of screening. Lung cancer–specific survival time conditioned on sex, age at diagnosis, histology, and stage is estimated by using cure models with lognormal survival distributions, which were fitted to lung cancer survival data in SEER 18.

The outputs from the natural history component serve as inputs for the screening component to simulate the effect of screening on lung cancer incidence, stage, and survival. The model uses sensitivity and specificity rates to simulate screening results: true positive, false positive, or negative. Sensitivity estimates were obtained from ten Haaf et al and vary by screening round, lung cancer histology, and stage [5]. These estimates, originally based on NLST, were adjusted to conform to Lung-RADS criteria by multiplying them by a scaling factor, given by the ratio of the overall sensitivity from Lung-RADS over that from NLST [17]. Specificity rates by screening round were also based on a retrospective analysis of NLST outcomes using the Lung-RADS criteria [17].

The model simulates additional screening outcomes such as the number of follow-up tests and potential complications based on NLST rates [6, 18]. The rates of specificity and sensitivity for screening simulation were chosen based on other published literature [5, 6, 18]. For true-positive cases, lung cancer–specific survival time and stage burden were updated given the stage and age at diagnosis.

A version of this model using an alternative dose-response model (Bach’s lung cancer risk model) was recently used to evaluate the preference-sensitivity of LDCT at different levels of underlying risk [19, 20].

**Lung Cancer Outcomes Simulation (LCOS) model**

The Lung Cancer Outcomes Simulation (LCOS) model is based on a natural history model of lung cancer that assumes exponential growth for the primary tumor and growth of metastasis proportional to the primary tumor growth [4, 21]. The natural history model simulates the individual’s tumor growth in the absence of any intervention, based on sex and histologic subtype, providing outcomes such as tumor volume doubling time, time for onset of metastasis, tumor size at clinical detection, and survival time. The parameters of the natural history model are estimated using SEER survival data.

In the natural history model, the primary tumor grows exponentially with growth rate r, and its corresponding tumor volume doubling time is given as (log2)/r. When the tumor reaches a certain size (Vp), it prompts symptoms that lead to a clinical detection of the primary tumor. The tumor volume and the growth rate parameter are modeled as a bivariate log-normal distribution. Fatal metastases start growing at a certain time, and we assume that the volume of the primary tumor at this time (Vc) is a threshold for cure (cure threshold) so that only if the primary tumor is detected and treated earlier than this point is the patient cured. The metastatic burden grows proportionally to the primary tumor size with fraction “f” until it reaches a maximum burden size (BD). The time at which BD is reached is a survival time if the primary tumor is not detected and treated before reaching the cure threshold. If this metastatic burden grows to a certain size (c1BD, where 0<c1<1), it becomes an observable metastasis; thus, if detection (either clinical or screen detection) occurs earlier than this point, the tumor is staged as early stage. Otherwise, the tumor is staged as advanced. When the metastatic burden grows to a certain size (c2BD, where 0<c2<1), it becomes clinically symptomatic; hence, detection is due to either the primary tumor or metastasis, depending on which becomes symptomatic first.

LCOS superimposes a specific screening intervention to each individual and estimates individual-level outcomes, which can be aggregated for evaluating population-level outcomes. Key inputs for LCOS include sex, individual-level smoking history (e.g., pack-years and age for starting/quitting smoking), and age of entry to the screening program. LCOS uses the TSCE model to predict annual hazards for lung cancer incidence in the absence of screening given smoking history, sex, and age [3]. The parameters of the TSCE model were estimated based on data from Nurses’ Health Study (NHS)/Health Professionals’ Follow-up Study (HPFS) with further adjustments [22].

For each lung cancer, a histologic subtype (adenocarcinoma, squamous, large cell, or small cell) is assigned by sampling from the observed proportions from SEER data. The lung cancer cases with indeterminate diagnosis follow the Lung-RADS guidelines, which are explicitly incorporated in LCOS [22]. LCOS compares the performance of the screening program of interest against the no-screen scenario estimating the benefit of screening. It can estimate sex-specific benefits and harms of screening strategies with various starting and stopping screening ages, smoking exposure levels, screening frequencies, and follow-up protocols. Outcome measures include lung cancer deaths avoided, all-cause and lung cancer–specific mortality reduction, number of detected cases by mode of detection, number of screening examinations, number of false-positive results, and number of overdiagnosed cases, among others.

**Lung Cancer Policy Model (LCPM)**

The Lung Cancer Policy Model (LCPM) is a comprehensive state-transition microsimulation model that simulates a patient’s lung cancer development, progression, detection, follow-up, treatment, and survival [8, 23-31]. LCPM was originally developed to evaluate the clinical effectiveness and cost-effectiveness of low-dose computed tomography (LDCT) screening for lung cancer [26, 27, 29]. The model has been extended to estimate the effect of reduced tobacco smoking on lung cancer mortality in the United States, the number of radiation-induced cancers from lung cancer screening, cost-effectiveness of follow-up of incidentally detected pulmonary nodules, and effectiveness of treatment strategies for stage IA/IB non-small cell carcinoma patients who were non-operative candidates [8, 24, 25, 30, 31]. The model was used in the 2013 and 2021 CISNET lung cancer decision analysis for the USPSTF [8, 9].

LCPM is a Monte Carlo microsimulation model coded in C++. The model initially populates with disease-free persons who then go through different health states according to monthly transition probabilities. In each monthly cycle, an individual may develop lung cancer, have an existing cancer grow, or develop symptoms or metastases. The risk of lung cancer is related to persons’ smoking history, which is updated monthly (the model also includes cancers in nonsmokers). Smoking exposure history is supplied by the SHG. Lung cancers can be detected by an evaluation of symptoms through incidental imaging or by LDCT screening (with different tumor behavior for screen-detected cases). Persons with suspected lung cancer receive diagnostic and staging tests and then may undergo treatment. The screening module can be turned on and off to allow for analyses of treatment effectiveness for screen-detected vs. non-screen-detected cases.

Each simulated individual in LCPM can develop up to three cancers from any of five lung cancer cell types (adenocarcinoma, large cell, squamous cell, small cell, and other non-small cell carcinoma). For each cell type, the monthly probability of cancer development is described by a logistic equation with seven natural history parameters: a type-specific intercept, type-specific coefficients for age, age squared, years of cigarette exposure (pack-years), an interaction term between pack-years and age squared, the mean number of cigarettes smoked per day (cigarettes per day), and the years since quitting smoking. LCPM also explicitly simulates the follow-up schedule of small incidentally detected or screen-detected lung nodules, which allows the model to examine the effectiveness of follow-up recommendations by the Fleischner Society or the Lung Imaging Reporting and Data System (Lung-RADS).

In LCPM, the levels of simulated disease characteristics allow the staging of patients according to the Tumor Node Metastasis classification, permitting the model to simulate the most up-to-date treatment options according to current clinical practices [32]. The natural history parameters related to unobservable events (i.e., the initiation of the first cancer cell) were estimated by calibrating the model using Surveillance, Epidemiology, and End Results (SEER) registry data (cancer incidence by cell type, stage distribution at diagnosis, and stage-specific survival), published cohort studies, and clinical trial data. The details of model calibration and validation of the original natural history parameters have been described in previous publications [4, 33].

LCPM’s flexibility allows for evaluation of multiple components in a lung cancer screening program, including screening eligibility, smoking cessation, follow-up, staging, and treatment. Improvements in any of these components may affect patient outcomes and may influence the effectiveness of interventions in other areas. LCPM does not rely on data from a single trial to inform the parameter estimates but rather incorporates data from multiple sources and can incorporate new data as they emerge. LCPM can thus be used to evaluate screening in populations not included in ongoing trials and can address the “moving target” problem of improved test performance or treatment effectiveness.

**Oncosim**

Oncosim is a mathematical microsimulation model that simulates the smoking behavior of the Canadian population, and the impact of smoking behavior and exposure to radon on the incidence of lung cancer, healthcare costs, and all-cause deaths over an individual's lifetime.[34, 35]

Oncosim simulates smoking history of the Canadian population using a method similar to the Smoking History Generator of the other CISNET models. Specifically, Oncosim simulates the smoking patterns of the Canadian population, including smoking initiation rates, quit rates, and smoking intensity, which are derived from 3 large health surveys conducted in Canada (1979 Canada Health Survey, 1994–1995 National Population Health Survey, and Canadian Community Health Survey) [36-38]. Oncosim models the changes in smoking rates and intensity across time periods for each age group, by sex and province. Smoking trajectories were externally validated against other survey years and tobacco manufacturers’ data [39, 40].

The model calculates the annual risk of developing lung cancer for each person based on their smoking and radon exposure in two steps. First, the model calculates the risk of lung cancer using the age and smoking-related coefficients in the PLCOall2014 risk equation, which was developed from the control group (never and ever-smokers) in the Prostate, Lung, Colorectal and Ovarian Cancer Screening Trial (PLCO) and validated using data from the PLCO intervention arm [41]. Then, the model estimates the impact of radon exposure on lung cancer risk using a risk equation developed from a cohort study of uranium miners [42]. The resulting overall risk equation has a residual term that takes into account the effects of other factors (i.e., factors that are not related to age, smoking and radon exposure) on lung cancer risk. The residual term was calibrated to match the lung cancer incidence data from the 2017 Canadian Cancer Registry by age, sex and province.

For each incident lung cancer case, a histologic subtype (non-small cell lung cancer (NSCLC) or small cell lung cancer (SCLC​)) is assigned. The stage distribution of invasive NSCLC and SCLC varies by sex, age group, and jurisdiction, and is based on Canadian Cancer Registry data between 2010 and 2014. Oncosim uses a two-piece Weibull survival model that has been fit to Kaplan-Meier survival curves based on survival data from a cohort of individuals diagnosed with lung cancer in Ontario, Canada, with up to 3 years of follow-up data. All-cause survival parameters were estimated by stage and first treatment (surgery, chemotherapy, radiation therapy) and subsequently adjusted to align with age, sex, and provincial differences from the death-cleared Canadian Cancer Registry data. The calibration process also identified adjustment factors to simulate cause-specific survival of lung cancer diagnosed cases within the competing risk framework of other cause mortality in Oncosim.

The screening module includes eligibility criteria, diagnostic procedures for positive screens, and screening-related factors such as stage shift, sensitivity and specificity of low-dose CT [6]. Similar to other CISNET models, Oncosim estimates the impact of screening using a pre-clinical sojourn time. It models sojourn time using an exponential distribution and an overall sojourn time for all cancer stages. As the individual ages, the model compares the assigned detectable preclinical cancer phase with the time to clinical detection to determine the possibility of detectability by screening. The pre-clinical sojourn time and the sensitivity of screening were estimated to match the National Lung Screening Trial results [35].

The model simulates a variety of screening outcomes, such as the number of individuals eligible for screening, total scans, and the screening results. The model uses sensitivity and specificity rates to simulate screening results: true positive, false positive, or negative. The specificity was derived directly from NLST data. The sensitivity of screening was estimated through model fitting to match NLST incidence. The model simulates additional screening outcomes such as follow-up tests and incidental findings. The distribution of incidental findings was derived from the Dutch-Belgian lung cancer screening trial (NELSON) [43]. Oncosim also generates various other model outputs in the presence and absence of screening, such as the mortality due to lung cancer and other causes, overdiagnosis, life-years gained, and related healthcare costs.

**References**

[1] Jeon J, Meza R, Krapcho M, Clarke LD, Byrne J, Levy DT. Chapter 5: Actual and Counterfactual Smoking Prevalence Rates in the U.S. Population via Microsimulation. Risk Analysis. 2012; 32(s1):S51-S68.

[2] Hazelton WD, Jeon J, Meza R, Moolgavkar SH. Chapter 8: The FHCRC Lung Cancer Model. Risk Analysis. 2012; 32(s1):S99-S116.

[3] Meza R, Hazelton WD, Colditz GA, Moolgavkar SH. Analysis of lung cancer incidence in the nurses’ health and the health professionals’ follow-up studies using a multistage carcinogenesis model. Cancer Causes & Control. 2008; 19(3):317-28.

[4] Meza R, ten Haaf K, Kong CY, Erdogan A, Black WC, Tammemagi MC, et al. Comparative analysis of 5 lung cancer natural history and screening models that reproduce outcomes of the NLST and PLCO trials. Cancer. 2014; 120(11):1713-24.

[5] Ten Haaf K, van Rosmalen J, de Koning HJ. Lung cancer detectability by test, histology, stage, and gender: estimates from the NLST and the PLCO trials. Cancer Epidemiol Biomarkers Prev. 2015; 24(1):154-61.

[6] Reduced Lung-Cancer Mortality with Low-Dose Computed Tomographic Screening. New England Journal of Medicine. 2011; 365(5):395-409.

[7] Oken MM, Hocking WG, Kvale PA, Andriole GL, Buys SS, Church TR, et al. Screening by Chest Radiograph and Lung Cancer Mortality: The Prostate, Lung, Colorectal, and Ovarian (PLCO) Randomized Trial. JAMA. 2011; 306(17):1865-73.

[8] de Koning HJ, Meza R, Plevritis SK, ten Haaf K, Munshi VN, Jeon J, et al. Benefits and Harms of Computed Tomography Lung Cancer Screening Strategies: A Comparative Modeling Study for the U.S. Preventive Services Task Force. Annals of Internal Medicine. 2014; 160(5):311-20.

[9] Meza R, Jeon J, Toumazis I, ten Haaf K, Cao P, Bastani M, et al. Evaluation of the Benefits and Harms of Lung Cancer Screening With Low-Dose Computed Tomography: Modeling Study for the US Preventive Services Task Force. JAMA. 2021; 325(10):988-97.

[10] Cancer Australia. Report on the Lung Cancer Screening Enquiry. Surry Hills, NSW: Cancer Australia; 2020.

[11] Tomonaga Y, Ten Haaf K, Frauenfelder T, Kohler M, Kouyos RD, Shilaih M, et al. Cost-effectiveness of low-dose CT screening for lung cancer in a European country with high prevalence of smoking-A modelling study. Lung Cancer. 2018; 121:61-9.

[12] ten Haaf K, Tammemägi MC, Bondy SJ, van der Aalst CM, Gu S, McGregor SE, et al. Performance and Cost-Effectiveness of Computed Tomography Lung Cancer Screening Scenarios in a Population-Based Setting: A Microsimulation Modeling Analysis in Ontario, Canada. PLOS Medicine. 2017; 14(2):e1002225.

[13] Ten Haaf K, Bastani M, Cao P, Jeon J, Toumazis I, Han SS, et al. A Comparative Modeling Analysis of Risk-Based Lung Cancer Screening Strategies. J Natl Cancer Inst. 2020; 112(5):466-79.

[14] Criss SD, Cao P, Bastani M, Ten Haaf K, Chen Y, Sheehan DF, et al. Cost-Effectiveness Analysis of Lung Cancer Screening in the United States: A Comparative Modeling Study. Ann Intern Med. 2019; 171(11):796-804.

[15] Toumazis I, de Nijs K, Cao P, Bastani M, Munshi V, Ten Haaf K, et al. Cost-effectiveness Evaluation of the 2021 US Preventive Services Task Force Recommendation for Lung Cancer Screening. JAMA Oncol. 2021; 7(12):1833-42.

[16] Toumazis I, Cao P, de Nijs K, Bastani M, Munshi V, Hemmati M, et al. Risk Model-Based Lung Cancer Screening : A Cost-Effectiveness Analysis. Ann Intern Med. 2023; 176(3):320-32.

[17] Pinsky PF, Gierada DS, Black W, Munden R, Nath H, Aberle D, et al. Performance of Lung-RADS in the National Lung Screening Trial. Annals of Internal Medicine. 2015; 162(7):485-91.

[18] Aberle DR, DeMello S, Berg CD, Black WC, Brewer B, Church TR, et al. Results of the Two Incidence Screenings in the National Lung Screening Trial. New England Journal of Medicine. 2013; 369(10):920-31.

[19] Caverly TJ, Cao P, Hayward RA, Meza R. Identifying Patients for Whom Lung Cancer Screening Is Preference-Sensitive. Annals of Internal Medicine. 2018; 169(1):1-9.

[20] Bach PB, Kattan MW, Thornquist MD, Kris MG, Tate RC, Barnett MJ, et al. Variations in lung cancer risk among smokers. J Natl Cancer Inst. 2003; 95(6):470-8.

[21] Lin RS, Plevritis SK. Comparing the benefits of screening for breast cancer and lung cancer using a novel natural history model. Cancer Causes & Control. 2012; 23(1):175-85.

[22] Han SS, Erdogan SA, Toumazis I, Leung A, Plevritis SK. Evaluating the impact of varied compliance to lung cancer screening recommendations using a microsimulation model. Cancer Causes & Control. 2017; 28(9):947-58.

[23] McMahon PM, Meza R, Plevritis SK, Black WC, Tammemagi CM, Erdogan A, et al. Comparing Benefits from Many Possible Computed Tomography Lung Cancer Screening Programs: Extrapolating from the National Lung Screening Trial Using Comparative Modeling. PLOS ONE. 2014; 9(6):e99978.

[24] Goehler A, McMahon PM, Lumish HS, Wu CC, Munshi V, Gilmore M, et al. Cost-effectiveness of follow-up of pulmonary nodules incidentally detected on cardiac computed tomographic angiography in patients with suspected coronary artery disease. Circulation. 2014; 130(8):668-75.

[25] Kong CY, Lee JM, McMahon PM, Lowry KP, Omer ZB, Eisenberg JD, et al. Using Radiation Risk Models in Cancer Screening Simulations: Important Assumptions and Effects on Outcome Projections. Radiology. 2012; 262(3):977-84.

[26] McMahon PM, Kong CY, Bouzan C, Weinstein MC, Cipriano LE, Tramontano AC, et al. Cost-effectiveness of computed tomography screening for lung cancer in the United States. J Thorac Oncol. 2011; 6(11):1841-8.

[27] McMahon PM, Kong CY, Johnson BE, Weinstein MC, Weeks JC, Kuntz KM, et al. Estimating Long-term Effectiveness of Lung Cancer Screening in the Mayo CT Screening Study. Radiology. 2008; 248(1):278-87.

[28] McMahon PM, Kong CY, Johnson BE, Weinstein MC, Weeks JC, Tramontano AC, et al. Chapter 9: The MGH-HMS Lung Cancer Policy Model: Tobacco Control Versus Screening. Risk Analysis. 2012; 32(s1):S117-S24.

[29] McMahon PM, Kong CY, Weinstein MC, Tramontano AC, Cipriano LE, Johnson BE, et al. Adopting helical CT screening for lung cancer: potential health consequences during a 15-year period. Cancer. 2008; 113(12):3440-9.

[30] Moolgavkar SH, Holford TR, Levy DT, Kong CY, Foy M, Clarke L, et al. Impact of reduced tobacco smoking on lung cancer mortality in the United States during 1975-2000. J Natl Cancer Inst. 2012; 104(7):541-8.

[31] Angela CT, Deirdre FS, Pamela MM, Emily CD, Theodore RH, Karen R, et al. Evaluating the impacts of screening and smoking cessation programmes on lung cancer in a high-burden region of the USA: a simulation modelling study. BMJ Open. 2016; 6(2):e010227.

[32] Detterbeck FC, Boffa DJ, Kim AW, Tanoue LT. The Eighth Edition Lung Cancer Stage Classification. Chest. 2017; 151(1):193-203.

[33] Kong CY, McMahon PM, Gazelle GS. Calibration of Disease Simulation Model Using an Engineering Approach. Value in Health. 2009; 12(4):521-9.

[34] Evans WK, Wolfson M, Flanagan WM, Shin J, Goffin JR, Asakawa K, et al. The evaluation of cancer control interventions in lung cancer using the Canadian Cancer Risk Management Model. Lung Cancer Management. 2012; 1(1):25-33.

[35] Flanagan WM, Evans WK, Fitzgerald NR, Goffin JR, Miller AB, Wolfson MC. Performance of the cancer risk management model lung cancer screening module. Health Rep. 2015; 26(5):11-8.

[36] Statistics Canada. Canada Health Survey 1978/1979.

[37] Statistics Canada. National Population Health Survey (NPHS):1994-2010.

[38] Statistics Canada. Canadian Community Health Survey 2001-2017.

[39] Statistics Canada. Table 303-0062 - Production, sales and inventories of tobacco products, monthly (kilograms unless otherwise noted), CANSIM (database). Accessed 2014-10-07.

[40] Forey B, Hamling J, Hamling J, Lee P. International Smoking Statistics Web Edition Canada. Sutton, United Kingdom: P.N. Lee Statistics & Computing Ltd. 2009; <http://www.pnlee.co.uk/iss.htm>.

[41] Tammemägi MC, Church TR, Hocking WG, Silvestri GA, Kvale PA, Riley TL, et al. Evaluation of the Lung Cancer Risks at Which to Screen Ever- and Never-Smokers: Screening Rules Applied to the PLCO and NLST Cohorts. PLOS Medicine. 2014; 11(12):e1001764.

[42] Whittemore AS, McMillan A. Lung Cancer Mortality Among U.S. Uranium Miners: A Reappraisal2. JNCI: Journal of the National Cancer Institute. 1983; 71(3):489-99.

[43] van de Wiel JCM, Wang Y, Xu DM, van der Zaag-Loonen HJ, van der Jagt EJ, van Klaveren RJ, et al. Neglectable benefit of searching for incidental findings in the Dutch--Belgian lung cancer screening trial (NELSON) using low-dose multidetector CT. European Radiology. 2007; 17(6):1474-82.
