# Supplementary material for: The Impact of Model Assumptions on Personalized Lung Cancer Screening Recommendations
Source: Med Decis Making. 2024 May 13;44(5):497–511. doi: 10.1177/0272989X241249182 (PMC11281869; doi:10.1177/0272989X241249182)
Supplement: sj-docx-2-mdm-10.1177_0272989X241249182 – Supplemental material for The Impact of Model Assumptions on Personalized Lung Cancer Screening Recommendations [file sj-docx-2-mdm-10.1177_0272989X241249182.docx]

**Supplementary material**

**Supplementary Table 1: Key characteristics of model assumptions**

| **Model** | **Major assumptions or outcomes regarding sensitivity, sojourn time and treatment effectiveness of screen-detected cancers within a considered population affected** | **MISCAN** | **UOM/BCC** | **LCOS** | **LCPM** | **Oncosim** |
| --- | --- | --- | --- | --- | --- | --- |
| **Model type** | None | Microsimulation model, Parallel universe | Microsimulation model | Simulation, Parallel  universe | Microsimulation model | Microsimulation model |
| **Cycle length** | None | Continuous time | Continuous time | Continuous time | Monthly | Continuous time |
| **Type of smoking dose-response model** | None | Two-stage clonal expansion model (recalibrated to NLST, PLCO and SEER-17) | Two-stage clonal expansion model (recalibrated to US lung cancer mortality) | Two-stage clonal expansion model | Logistic model with coefficients for age, smoke-years, cigarettes per day, and years since quitting | The impact of smoking on lung cancer incidence is non-linear and acts through smoker type, intensity, and duration. |
| **Histological types considered** | Sensitivity, sojourn time, treatment effectiveness of screen-detected cancers | Adenocarcinoma (including BAC and large cell carcinoma), squamous cell carcinoma, other non-small cell carcinoma, small cell carcinoma | Adenocarcinoma (including BAC), squamous cell carcinoma, other non-small cell carcinoma, and small cell carcinoma | Adenocarcinoma (including BAC and large cell carcinoma), squamous cell carcinoma, small cell carcinoma | Adenocarcinoma, large cell carcinoma, small cell carcinoma, squamous cell carcinoma, other non-small cell carcinoma | Non-small cell lung cancer (NSCLC) and small cell lung cancer (SCLC​) |
| **Histological type assignment** | Sensitivity, sojourn time, treatment effectiveness of screen-detected cancers | Distribution of histologies differs by sex | Distribution of histologies is a function of sex, smoking status, cigarettes per day, smoking duration, and years since quit smoking | Distribution of histologies differs by sex | Distribution of histology differs by sex and smoking status | Stage distribution of invasive NSCLC and SCLC is based on Canadian Cancer Registry 2010 to 2014. |
| **Stage progression**  **modeled as** | Sensitivity, sojourn time | Stage-transition by  histology and sex | Stage-transition by  histology and sex | Continuous time growth of the primary tumor and metastases (as function of histology and sex) | Stage-transition by nodule histology and volume | Apart from recurrence (see below), stage progression is not modelled. |
| **Tumor stage information at diagnosis and screen-detection** | Treatment effectiveness of screen-detected cancers | Perfect | Perfect | TNM staging at diagnosis accounting for Lung-RADS guidelines on diagnostic follow up | Unknown at detection and imperfect at diagnosis (depending on sensitivity and specificity of diagnostic tests) | Perfect |
| **Tumor regression possible?** | Sojourn time | No | No | No | Yes | No |
| **Metastasis modeled separately?** | Sensitivity, sojourn time | No | No | Metastasis and primary tumor are modeled jointly | No | No |
| **Multiple cancers (synchronous/second) possible?** | Sensitivity, sojourn time | No | No | No (second cancer is possible, but not separately modeled in this analysis) | Up to three cancers and three benign nodules | Yes |
| **Screening sensitivity**  **dependent on** | Sensitivity | Stage and histology | Stage, histology and sex | Nodule size, sex and histology | Nodule diameter and location (central cancers have lower sensitivity) | Screening round, age group and NLST trial data; only NSCLC is detectable by screening in the model |
| **Tumor inception point** | Sensitivity, sojourn time | At the start of the preclinical screen-detectable phase | At the start of the preclinical screen-detectable phase | Primary tumors start at 1 mm3 | Inception at 0.01mm. For the purpose of this analysis, 1mm were considered relevant nodules. | At the start of the preclinical screen-detectable phase |
| **Lung cancer survival dependent on** | Treatment effectiveness of screen-detected cancers | Stage, histology and sex | Stage, histology, sex and age at diagnosis | Stage, histology and sex | Unsuccessful treatment progress to stage IV and dead. Survival depends on histology (NSCLC vs SCLC) and sex. | Weibull survival curves depend on cancer stage and treatment path. Further impacts on survival assessed through relative risks due to: screening, Canadian province, age, sex, treatment, and period effects. |
| **Recurrence modeled?** | Treatment effectiveness of screen-detected cancers | No, recurrence is included as part of the lung cancer survival process | No | Yes, but not included in this analysis | Recurrence modeled as part of the treatment component: unsuccessful treatment. | Yes (local and distant recurrence modelled separately) |
| **Implementation of screening benefit** | Treatment effectiveness of screen-detected cancers | Cure model* | Stage-shift model** | Cure model*** | Stage-shift model** | Screening results in stage shift and lower mortality risk (implemented through relative risk) |

**Table notes:** Abbreviations: Microsimulation Screening Analysis model (MISCAN); Lung Cancer Natural History and Screening model (UoM/BCC); Lung Cancer Outcomes Simulation model (LCOS); Lung Cancer Policy Model (LCPM).

* The benefit of screening is based on a stage-specific cure rate.

** The benefit of screening is based on a shift to a less advanced stage with the corresponding, more favorable stage-specific survival.

*** The probability of lethal metastases is estimated as a function of tumor size, histology and sex. With screening, patients are more likely to be detected at early stages and before the onset of lethal metastases, and cured following standard of care; patients are not cured if detected in early stages but after the onset of lethal metastases or in advanced stages.

**Supplementary Table 2: Screening sensitivity differences between models (heavy smokers, men versus women)**

| **Men** | | | | |
| --- | --- | --- | --- | --- |
| **Model** | **Stage I sensitivity** | **Stage II sensitivity** | **Stage III sensitivity** | **Stage IV sensitivity** |
| **MISCAN** | 38.2% | 42.2% | 70.9% | 98.0% |
| **UoM/BCC** | 30.1% | 33.4% | 62.3% | 86.1% |
| **LCOS** | 57.3% | 69.3% | 93.6% | 94.9% |
| **LCPM** | 43.1% | 66.5% | 90.3% | 86.6% |
| **Oncosim** | 91.9% | 91.1% | 94.1% | 91.2% |
| **Median** | 43.1% | 66.5% | 90.3% | 91.2% |
| Women | | | | |
| **Model** | **Stage I sensitivity** | **Stage II sensitivity** | **Stage III sensitivity** | **Stage IV sensitivity** |
| **MISCAN** | 41.9% | 45.3% | 71.7% | 98.1% |
| **UoM/BCC** | 32.3% | 36.5% | 63.0% | 87.8% |
| **LCOS** | 47.6% | 54.0% | 91.4% | 91.2% |
| **LCPM** | 43.8% | 67.4% | 90.0% | 86.3% |
| **Oncosim** | 91.2% | 90.5% | 89.0% | 91.6% |
| **Median** | 43.8% | 54.0% | 89.0% | 91.2% |

**Supplementary Table 3: Screening sensitivity differences between models (light smokers, men versus women)**

| **Men** | | | | |
| --- | --- | --- | --- | --- |
| **Model** | **Stage I sensitivity** | **Stage II sensitivity** | **Stage III sensitivity** | **Stage IV sensitivity** |
| **MISCAN** | 38.2% | 42.2% | 70.9% | 97.8% |
| **UoM/BCC** | 37.4% | 44.8% | 65.1% | 88.3% |
| **LCOS** | 53.2% | 69.3% | 97.7% | 94.6% |
| **LCPM** | 43.3% | 73.6% | 90.0% | 85.2% |
| **Oncosim** | 93.1% | 88.9% | 94.4% | 92.7% |
| **Median** | 43.3% | 69.3% | 90.0% | 92.7% |
| **Women** | | | | |
| **Model** | **Stage I sensitivity** | **Stage II sensitivity** | **Stage III sensitivity** | **Stage IV sensitivity** |
| **MISCAN** | 42.1% | 46.2% | 71.8% | 98.0% |
| **UoM/BCC** | 39.7% | 42.1% | 65.2% | 88.4% |
| **LCOS** | 41.6% | 52.2% | 90.4% | 89.1% |
| **LCPM** | 44.2% | 73.2% | 89.1% | 85.1% |
| **Oncosim** | 93.6% | 91.2% | 86.1% | 90.7% |
| **Median** | 42.1% | 52.2% | 86.1% | 89.1% |

**Supplementary Table 4: Screening effectiveness differences between models (heavy smokers, men versus women)**

| **Men** | | | | | |
| --- | --- | --- | --- | --- | --- |
|  | **All cancers leading to deaths detectable at screening** | | | **Screen-detected cases** | |
| **Model** | **Area A**  **(Death prevented)** | **Area B**  **(Detected but not prevented)** | **Area C**  **(Not detected, not prevented)** | **A / A+B**  **(detected and prevented)** | **B / A+B**  **(detected but not prevented)** |
| **MISCAN** | 23.0% | 25.1% | 51.9% | 47.8% | 52.2% |
| **UoM/BCC** | 10.0% | 26.9% | 63.1% | 27.1% | 72.9% |
| **LCOS** | 7.5% | 55.3% | 37.3% | 11.9% | 88.1% |
| **LCPM** | 20.7% | 46.3% | 33.0% | 30.9% | 69.1% |
| **Oncosim** | 23.6% | 71.3% | 5.1% | 24.9% | 75.1% |
| **Median** | 20.7% | 46.3% | 37.3% | 27.1% | 72.9% |
| **Women** | | | | | |
|  | **All cancers leading to deaths detectable at screening** | | | **Screen-detected cases** | |
| **Model** | **Area A**  **(Death prevented)** | **Area B**  **(Detected but not prevented)** | **Area C**  **(Not detected, not prevented)** | **A / A+B**  **(detected and prevented)** | **B / A+B**  **(detected but not prevented)** |
| **MISCAN** | 25.0% | 25.3% | 49.7% | 49.7% | 50.3% |
| **UoM/BCC** | 12.7% | 26.2% | 61.0% | 32.7% | 67.3% |
| **LCOS** | 9.5% | 45.3% | 45.1% | 17.4% | 82.6% |
| **LCPM** | 22.5% | 44.4% | 33.1% | 33.6% | 66.4% |
| **Oncosim** | 28.7% | 65.2% | 6.0% | 30.6% | 69.4% |
| **Median** | 22.5% | 44.4% | 45.1% | 32.7% | 67.3% |

**Supplementary Table 5: Screening effectiveness differences between models (light smokers, men versus women)**

| **Men** | | | | | |
| --- | --- | --- | --- | --- | --- |
|  | **All cancers leading to deaths detectable at screening** | | | **Screen-detected cases** | |
| **Model** | **Area A**  **(Death prevented)** | **Area B**  **(Detected but not prevented)** | **Area C**  **(Not detected, not prevented)** | **A / A+B**  **(detected and prevented)** | **B / A+B**  **(detected but not prevented)** |
| **MISCAN** | 22.5% | 26.0% | 51.5% | 46.4% | 53.6% |
| **UoM/BCC** | 12.8% | 31.6% | 55.6% | 28.9% | 71.1% |
| **LCOS** | 7.9% | 52.7% | 39.4% | 13.1% | 86.9% |
| **LCPM** | 21.9% | 44.4% | 33.7% | 33.0% | 67.0% |
| **Oncosim** | 10.9% | 84.6% | 4.5% | 11.4% | 88.6% |
| **Median** | 12.8% | 44.4% | 39.4% | 28.9% | 71.1% |
| **Women** | | | | | |
|  | **All cancers leading to deaths detectable at screening** | | | **Screen-detected cases** | |
| **Model** | **Area A**  **(Death prevented)** | **Area B**  **(Detected but not prevented)** | **Area C**  **(Not detected, not prevented)** | **A / A+B**  **(detected and prevented)** | **B / A+B**  **(detected but not prevented)** |
| **MISCAN** | 24.6% | 26.5% | 48.9% | 48.1% | 51.9% |
| **UoM/BCC** | 16.0% | 29.6% | 54.4% | 35.1% | 64.9% |
| **LCOS** | 9.4% | 43.1% | 47.5% | 17.9% | 82.1% |
| **LCPM** | 22.7% | 42.8% | 34.6% | 34.7% | 65.3% |
| **Oncosim** | 22.4% | 75.2% | 2.3% | 23.0% | 77.0% |
| **Median** | 22.4% | 42.8% | 47.5% | 34.7% | 65.3% |

**Supplementary Table 6: Screening sensitivity differences between models (heavy smokers versus light smokers)**

| **Heavy smokers** | | | | |
| --- | --- | --- | --- | --- |
| **Model** | **Stage I sensitivity** | **Stage II sensitivity** | **Stage III sensitivity** | **Stage IV sensitivity** |
| **MISCAN** | 40.3% | 43.9% | 71.3% | 98.0% |
| **UoM/BCC** | 31.3% | 35.1% | 62.7% | 87.1% |
| **LCOS** | 52.1% | 61.8% | 92.2% | 92.6% |
| **LCPM** | 43.5% | 67.0% | 90.2% | 86.4% |
| **Oncosim** | 91.5% | 90.8% | 91.2% | 91.4% |
| **Median** | 43.5% | 61.8% | 90.2% | 91.4% |
| **Light smokers** | | | | |
| **Model** | **Stage I sensitivity** | **Stage II sensitivity** | **Stage III sensitivity** | **Stage IV sensitivity** |
| **MISCAN** | 40.5% | 44.5% | 71.4% | 97.9% |
| **UoM/BCC** | 38.5% | 43.5% | 65.2% | 88.3% |
| **LCOS** | 46.8% | 60.5% | 93.2% | 91.4% |
| **LCPM** | 43.8% | 73.4% | 89.5% | 85.2% |
| **Oncosim** | 93.4% | 90.2% | 89.9% | 91.6% |
| **Median** | 43.8% | 60.5% | 89.5% | 91.4% |

**Supplementary Table 7: Screening effectiveness differences between models (heavy smokers versus light smokers)**

| **Heavy smokers** | | | | | |
| --- | --- | --- | --- | --- | --- |
|  | **All cancers leading to deaths detectable at screening** | | | **Screen-detected cases** | |
| **Model** | **Area A**  **(Death prevented)** | **Area B**  **(Detected but not prevented)** | **Area C**  **(Not detected, not prevented)** | **A / A+B**  **(detected and prevented)** | **B / A+B**  **(detected but not prevented)** |
| **MISCAN** | 24.1% | 25.2% | 50.7% | 48.9% | 51.1% |
| **UoM/BCC** | 11.5% | 26.5% | 62.0% | 30.2% | 69.8% |
| **LCOS** | 8.5% | 50.1% | 41.4% | 14.6% | 85.4% |
| **LCPM** | 21.7% | 45.2% | 33.1% | 32.5% | 67.5% |
| **Oncosim** | 26.4% | 68.0% | 5.6% | 28.0% | 72.0% |
| **Median** | 21.7% | 45.2% | 41.4% | 30.2% | 69.8% |
| **Light smokers** | | | | | |
|  | **All cancers leading to deaths detectable at screening** | | | **Screen-detected cases** | |
| **Model** | **Area A**  **(Death prevented)** | **Area B**  **(Detected but not prevented)** | **Area C**  **(Not detected, not prevented)** | **A / A+B**  **(detected and prevented)** | **B / A+B**  **(detected but not prevented)** |
| **MISCAN** | 23.7% | 26.3% | 50.0% | 47.4% | 52.6% |
| **UoM/BCC** | 14.3% | 30.6% | 55.0% | 31.9% | 68.1% |
| **LCOS** | 8.7% | 47.8% | 43.5% | 15.4% | 84.6% |
| **LCPM** | 22.3% | 43.5% | 34.2% | 33.9% | 66.1% |
| **Oncosim** | 17.6% | 79.2% | 3.2% | 18.2% | 81.8% |
| **Median** | 17.6% | 43.5% | 43.5% | 31.9% | 68.1% |

**Supplementary Figure 1: General overview of the structure of a natural-history model**


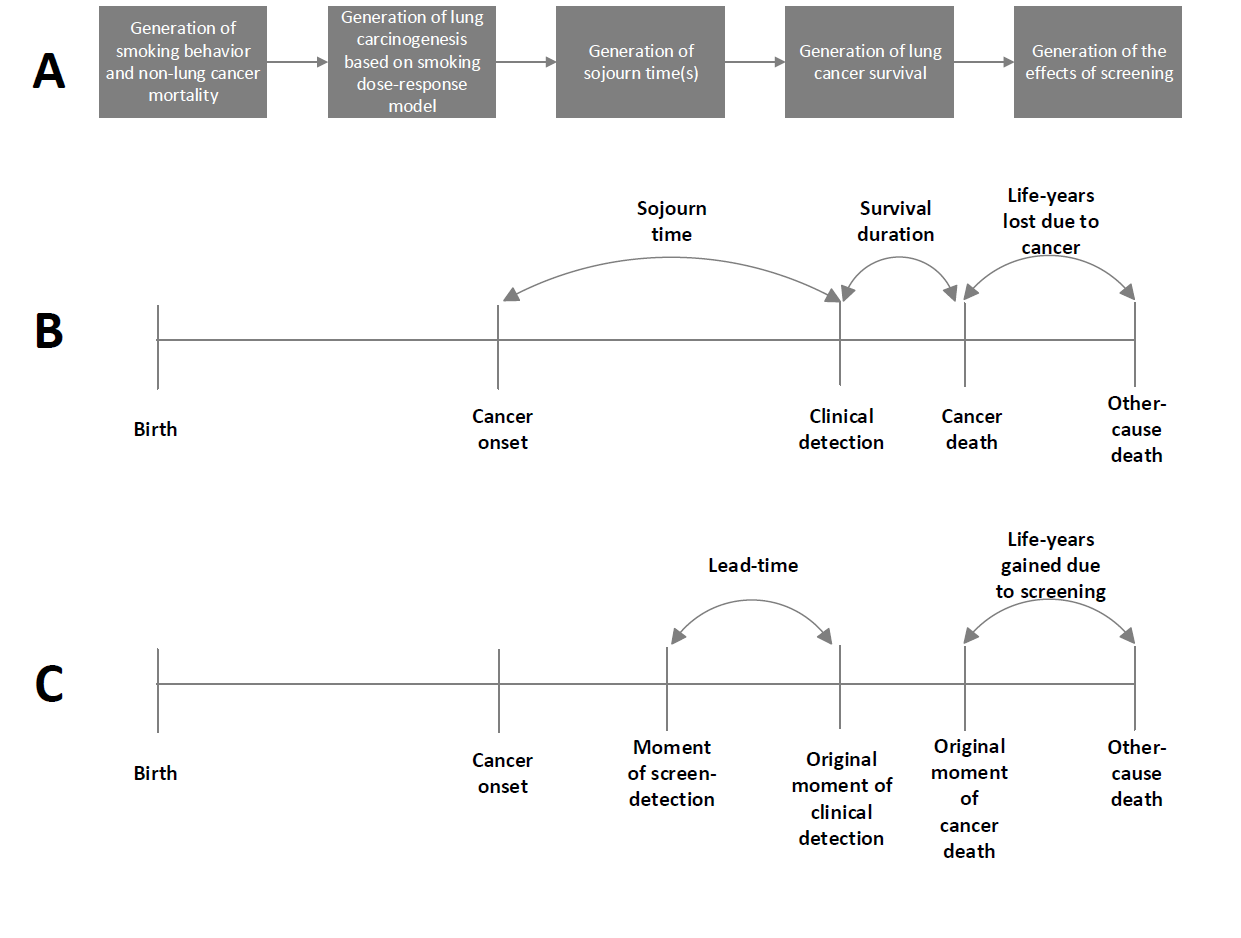


**Figure notes:**

Part A of the Figure provides a general overview of the natural-history models evaluated in this analysis.

Part B of the Figure provides an overview of a life-history of an individual in a model in the absence of screening.

Part C of the Figure provides an overview of the life-history of the individual in Part B in the presence of screening. In this case, screening is assumed to detect the cancer during the preclinical phase. Furthermore, screen-detection is assumed to lead to improved treatment options, allowing the patient to survive longer, until they die of other-causes.

**Supplementary Figure 2: stage distributions in the absence of screening (heavy smokers, both sexes combined)**

**
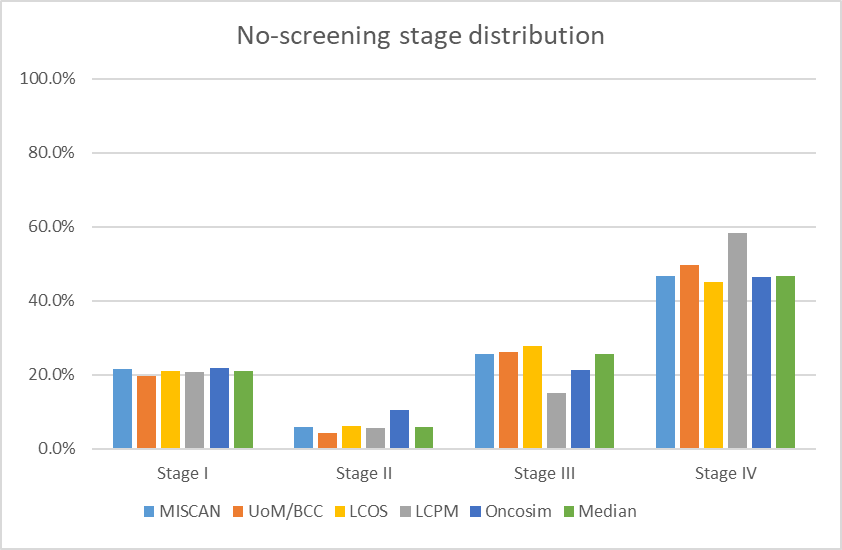
**

**Supplementary Figure 3: stage distributions of screen-detected cases under perfect sensitivity (heavy smokers, both sexes combined)**

**
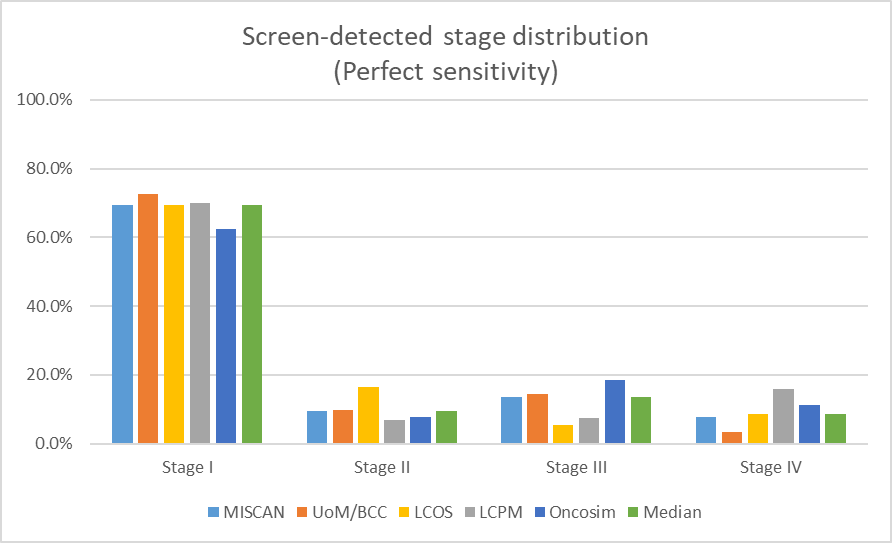
**

**Supplementary Figure 4: 15-year model-specific lung cancer incidence reductions under different assumptions**

**(heavy smokers, men versus women)**

**Men Women**

**
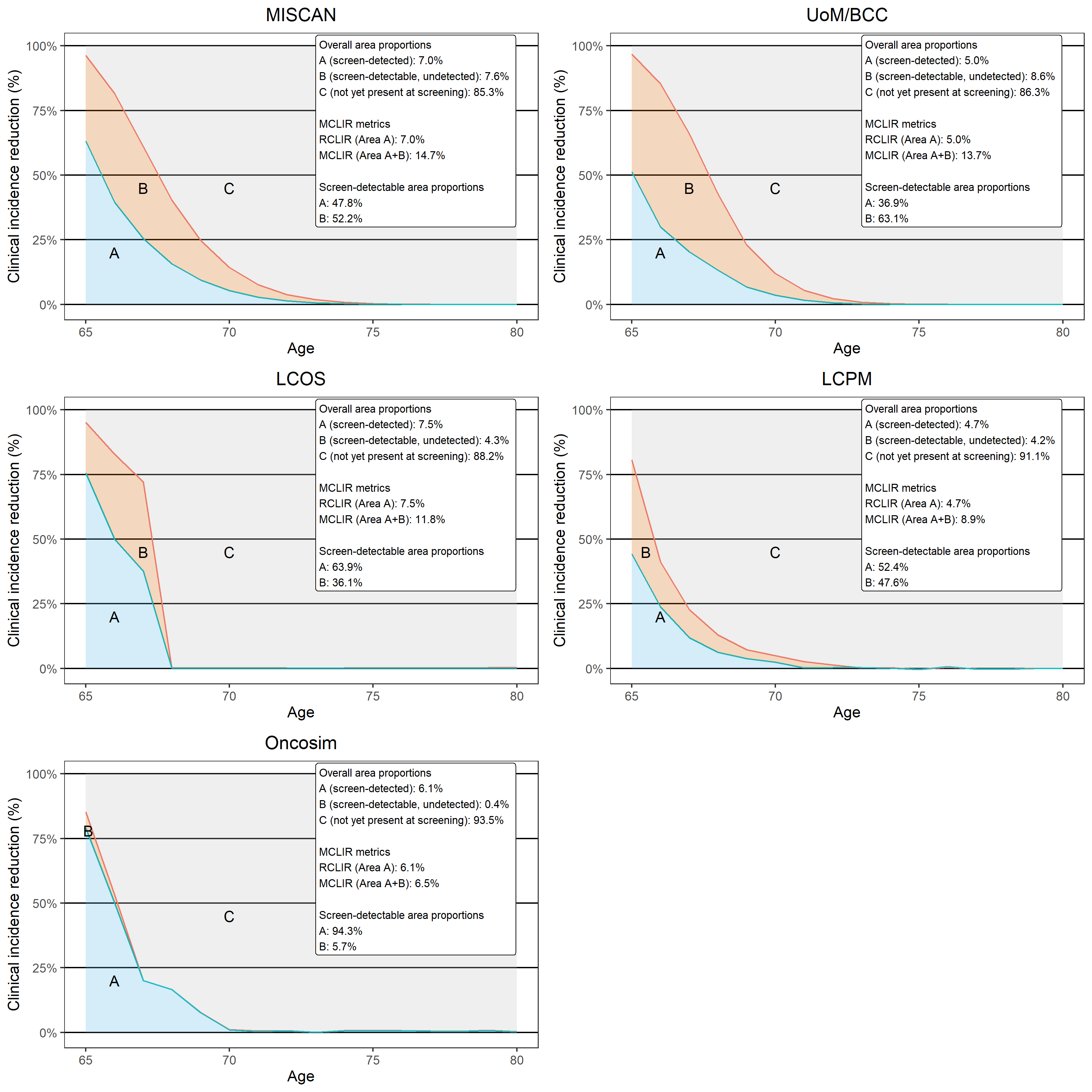

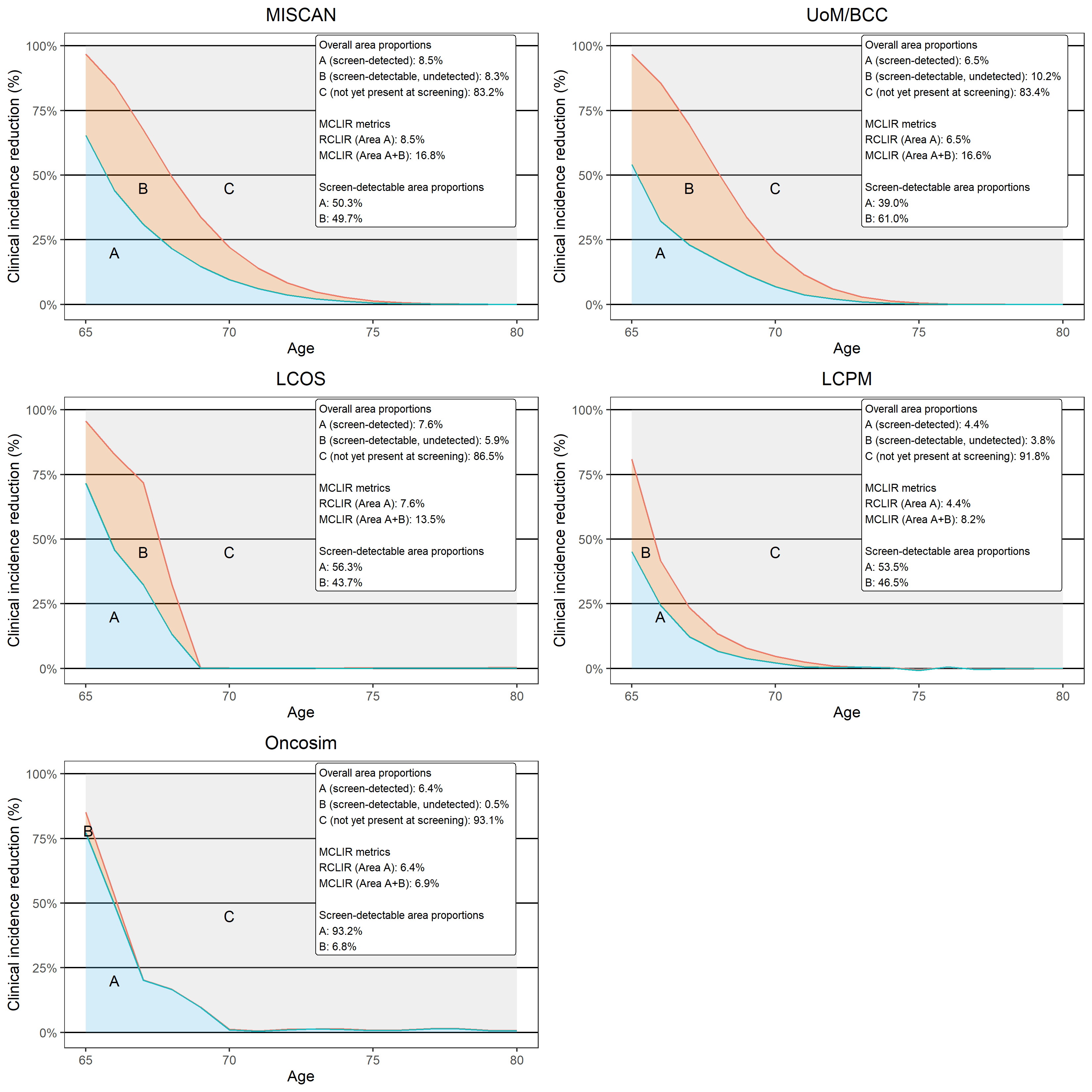
**

**Supplementary Figure 5: 15-year model-specific lung cancer incidence reductions under different assumptions**

**(light smokers, men versus women)**

**Men Women**

**
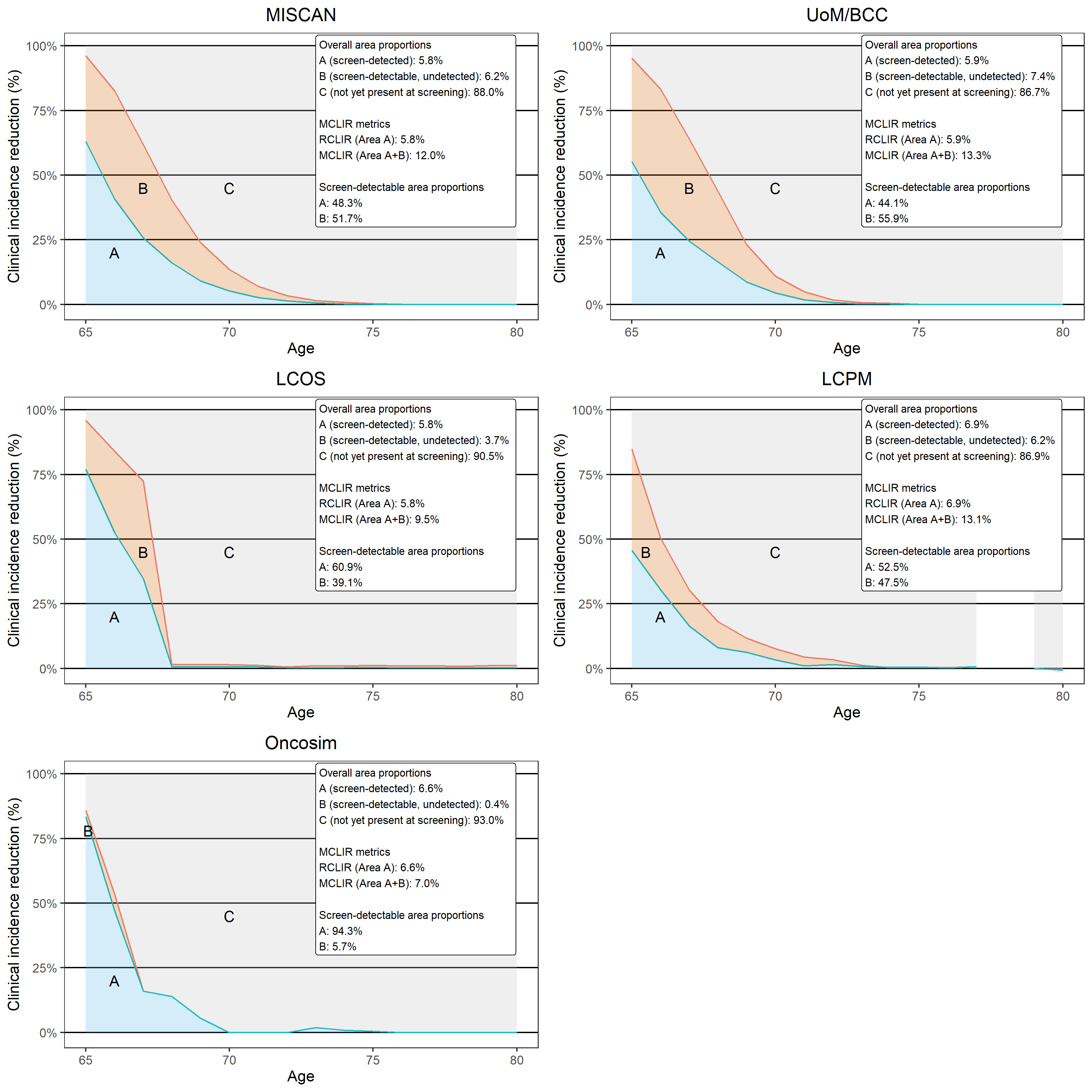

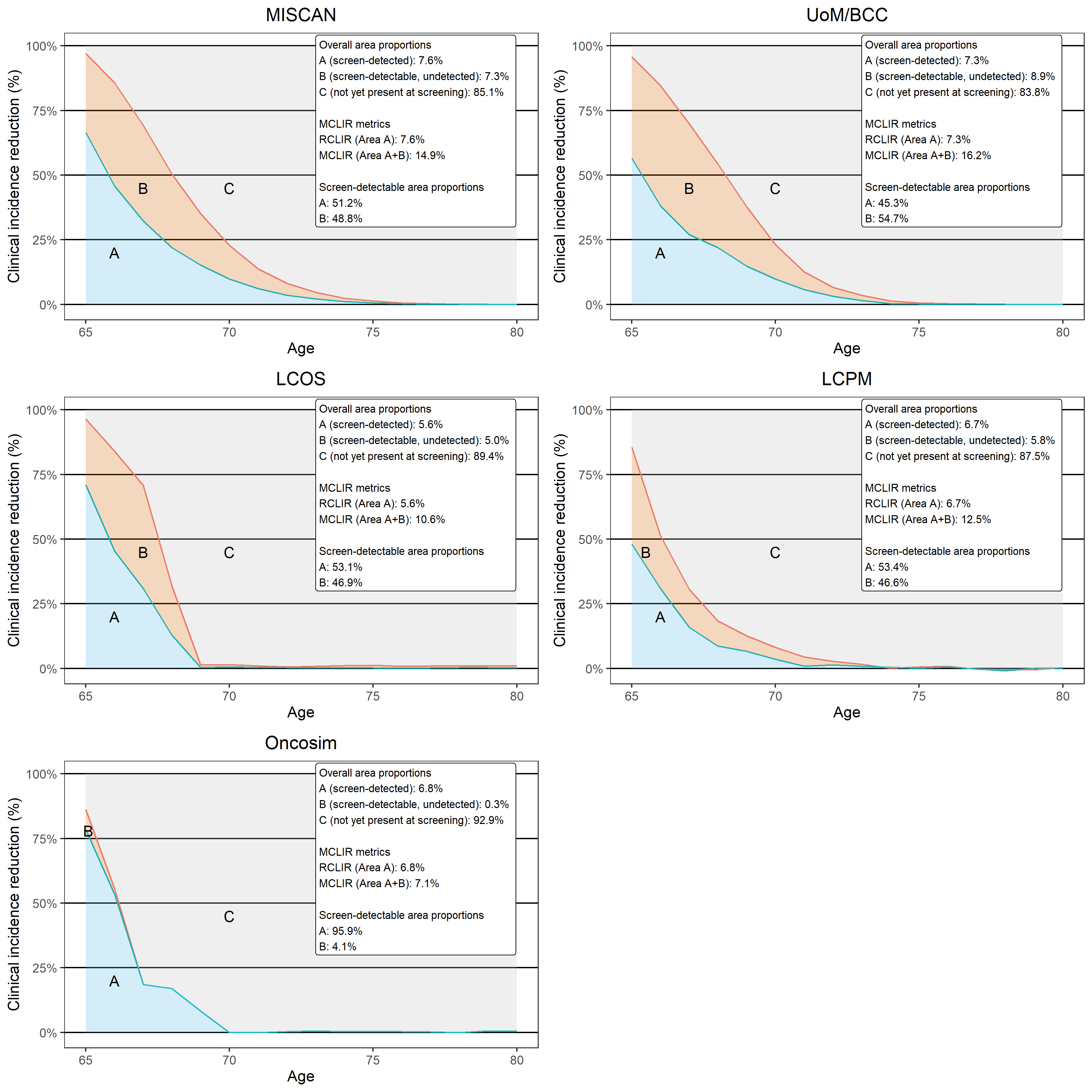
**

**Supplementary Figure 6: stage distributions in the absence of screening (heavy smokers, men versus women)**

**Men Women**

**
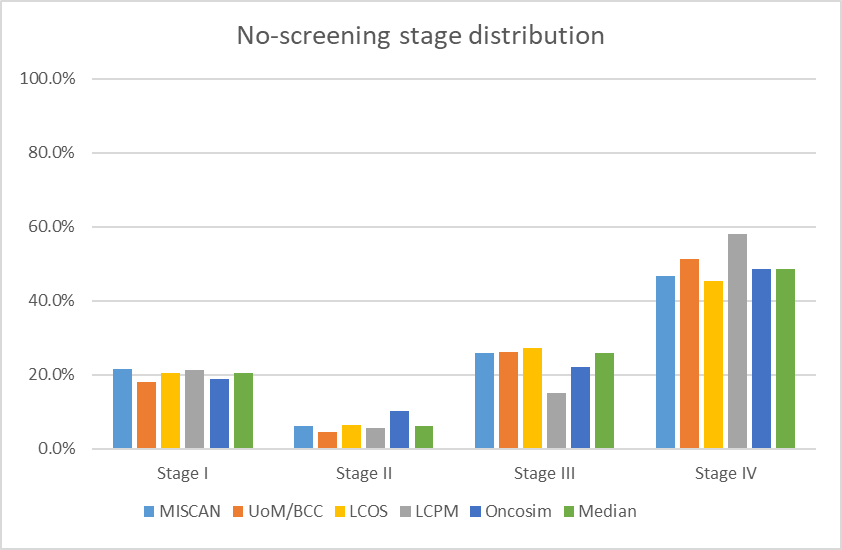

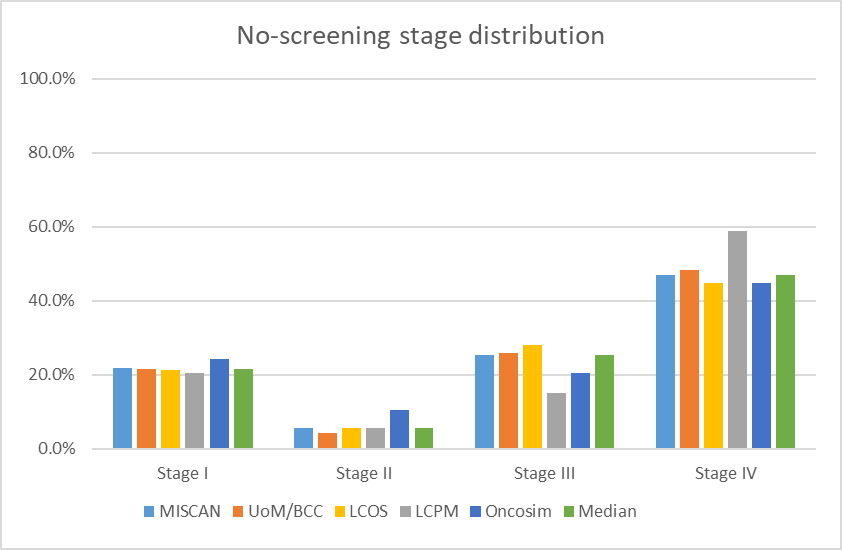
**

**Supplementary Figure 7: stage distributions in the absence of screening (light smokers, men versus women)**

**Men Women**

**
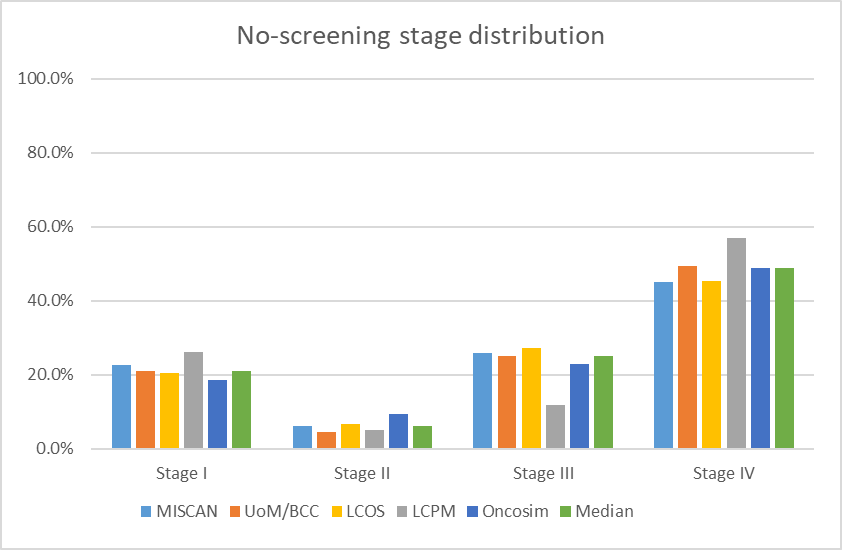

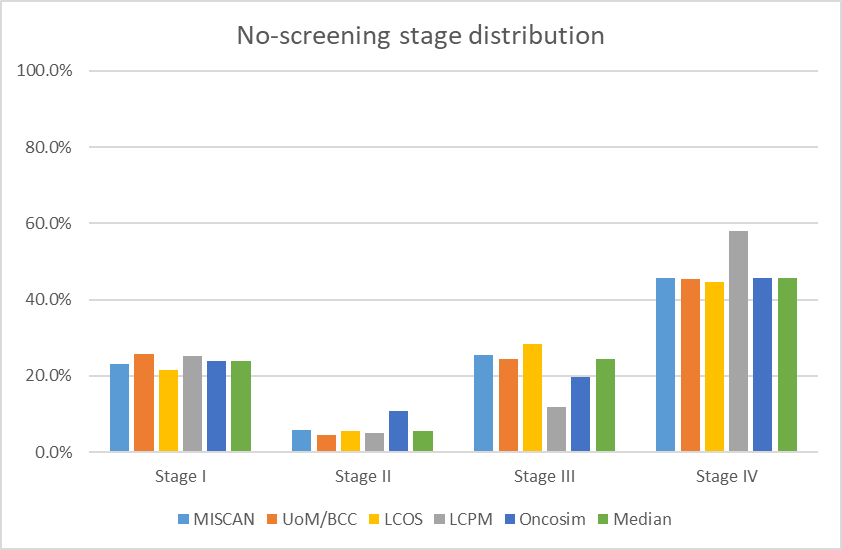
**

**Supplementary Figure 8: stage distributions of screen-detected cases under perfect sensitivity (heavy smokers, men versus women)**

**Men Women**

**
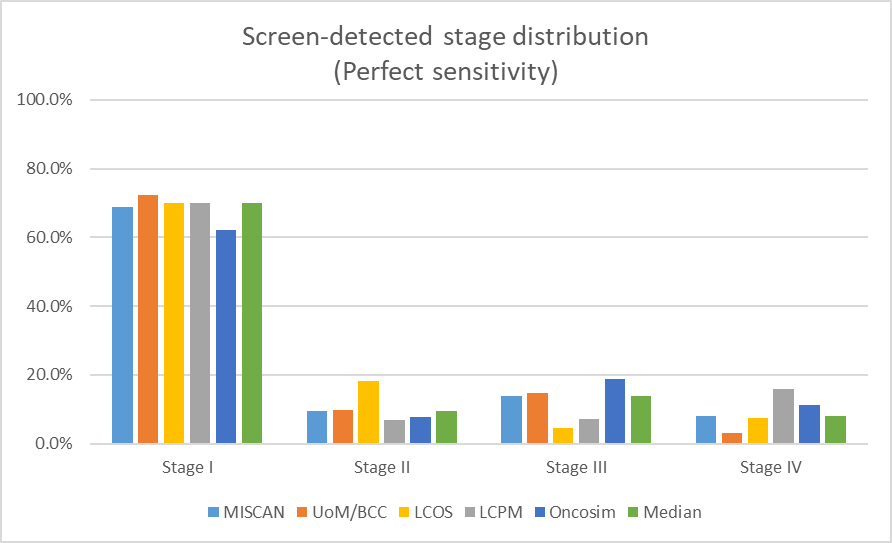

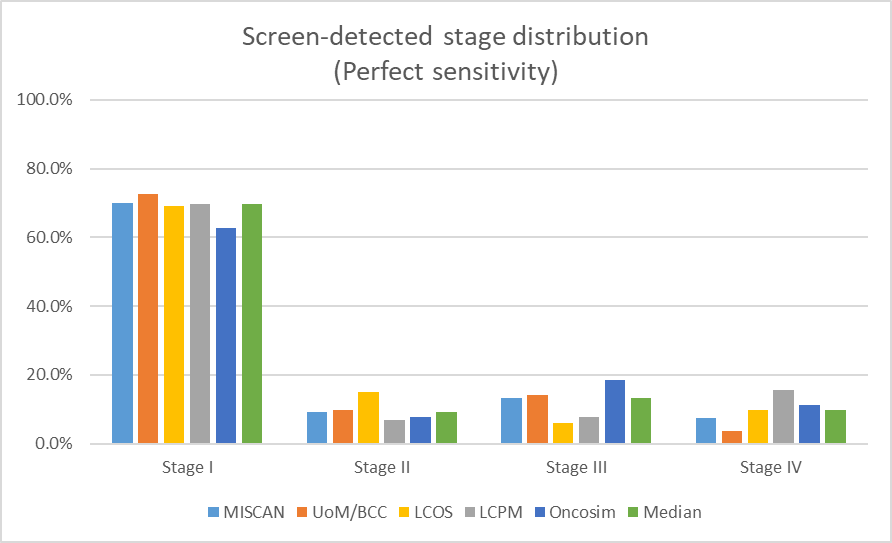
**

**Supplementary Figure 9: stage distributions of screen-detected cases under perfect sensitivity (light smokers, men versus women)**

**Men Women**

**
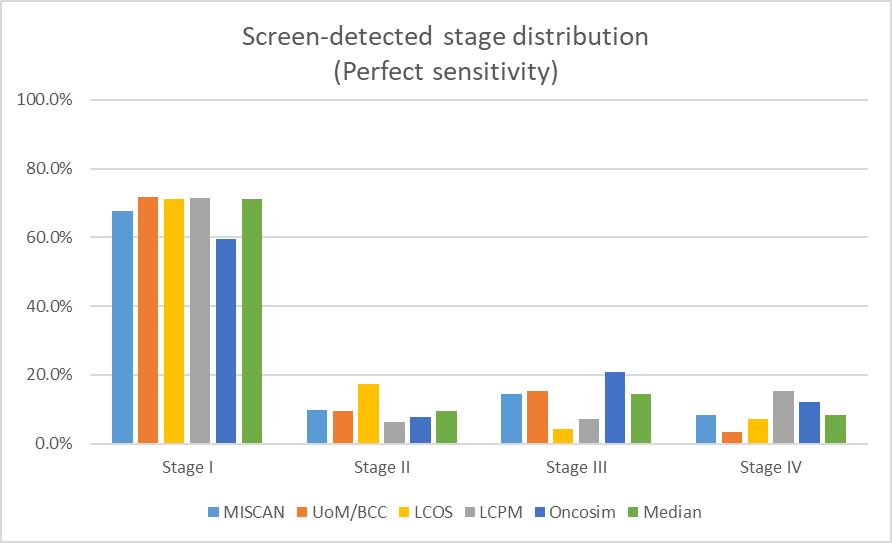

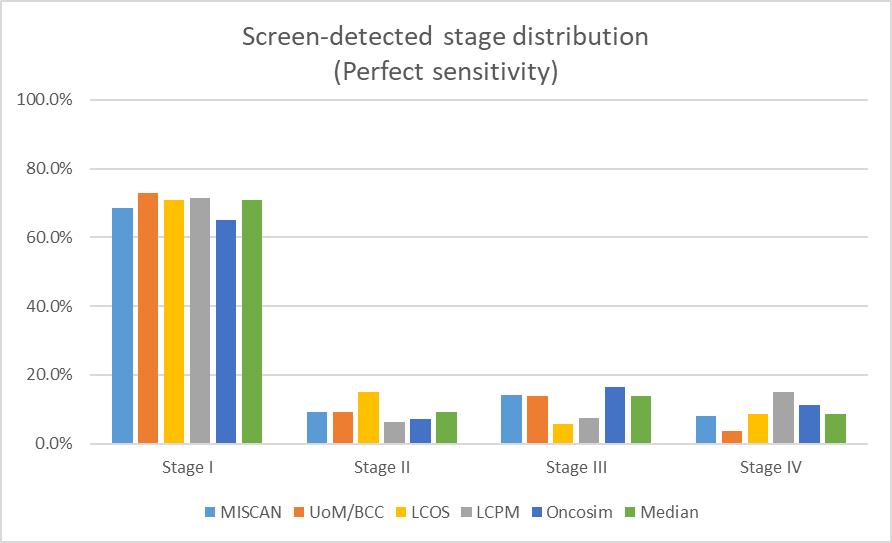
**

**Supplementary Figure 10: The effect of sensitivity on the occurrence of stage IV cancers post-screening (heavy smokers, men versus women)**

**Men Women**

**
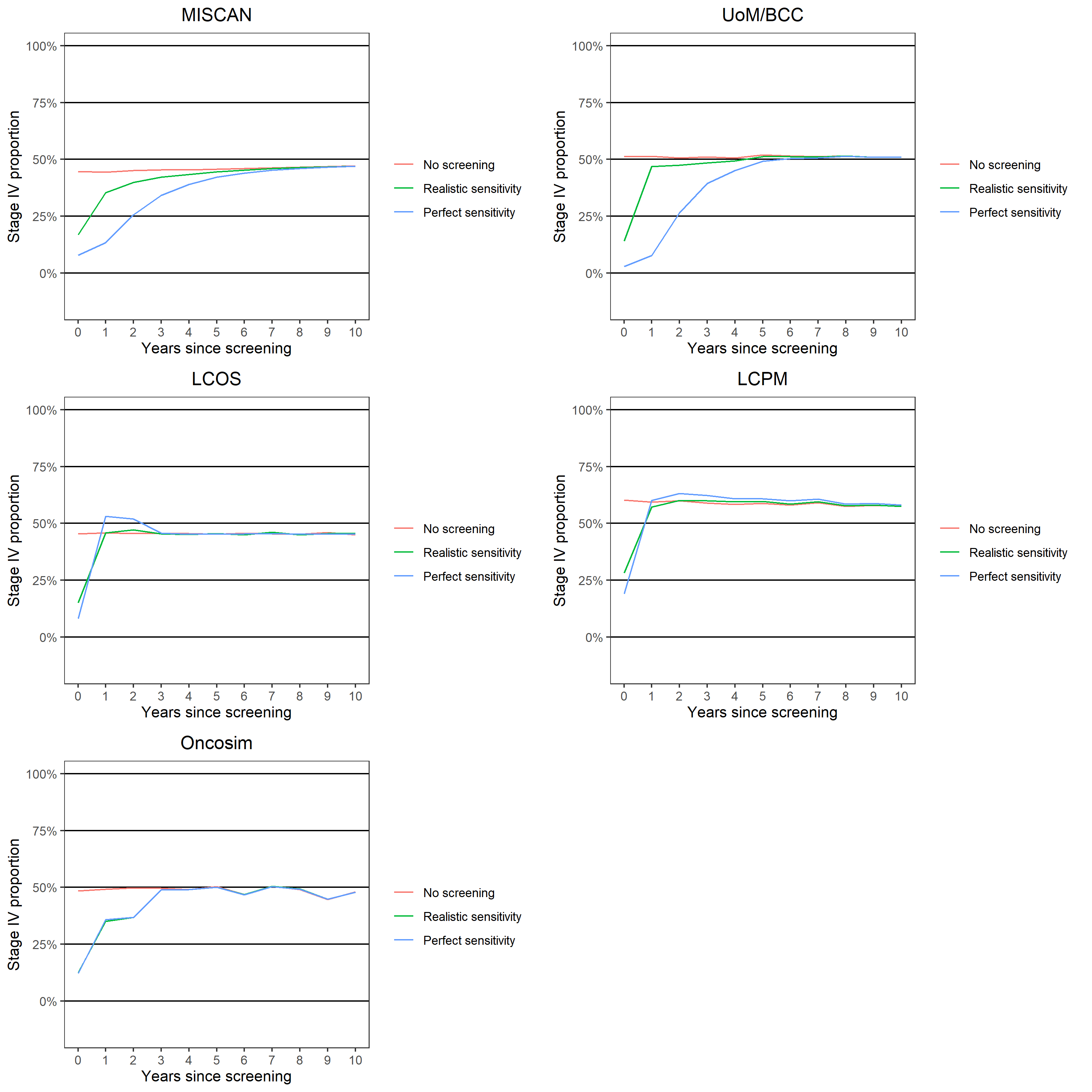

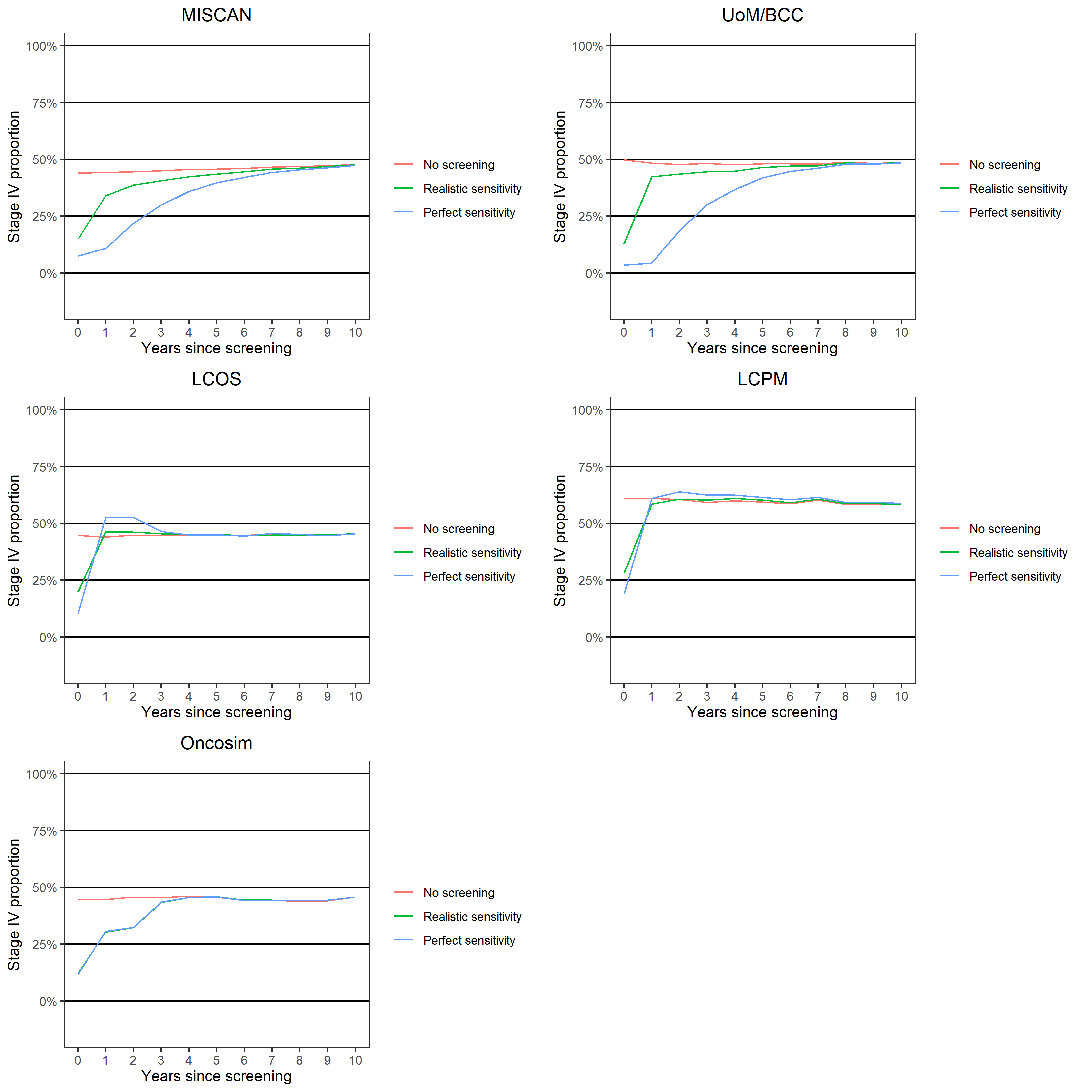
**

**Supplementary Figure 11: The effect of sensitivity on the occurrence of stage IV cancers post-screening (light smokers, men versus women)**

**Men Women**

**
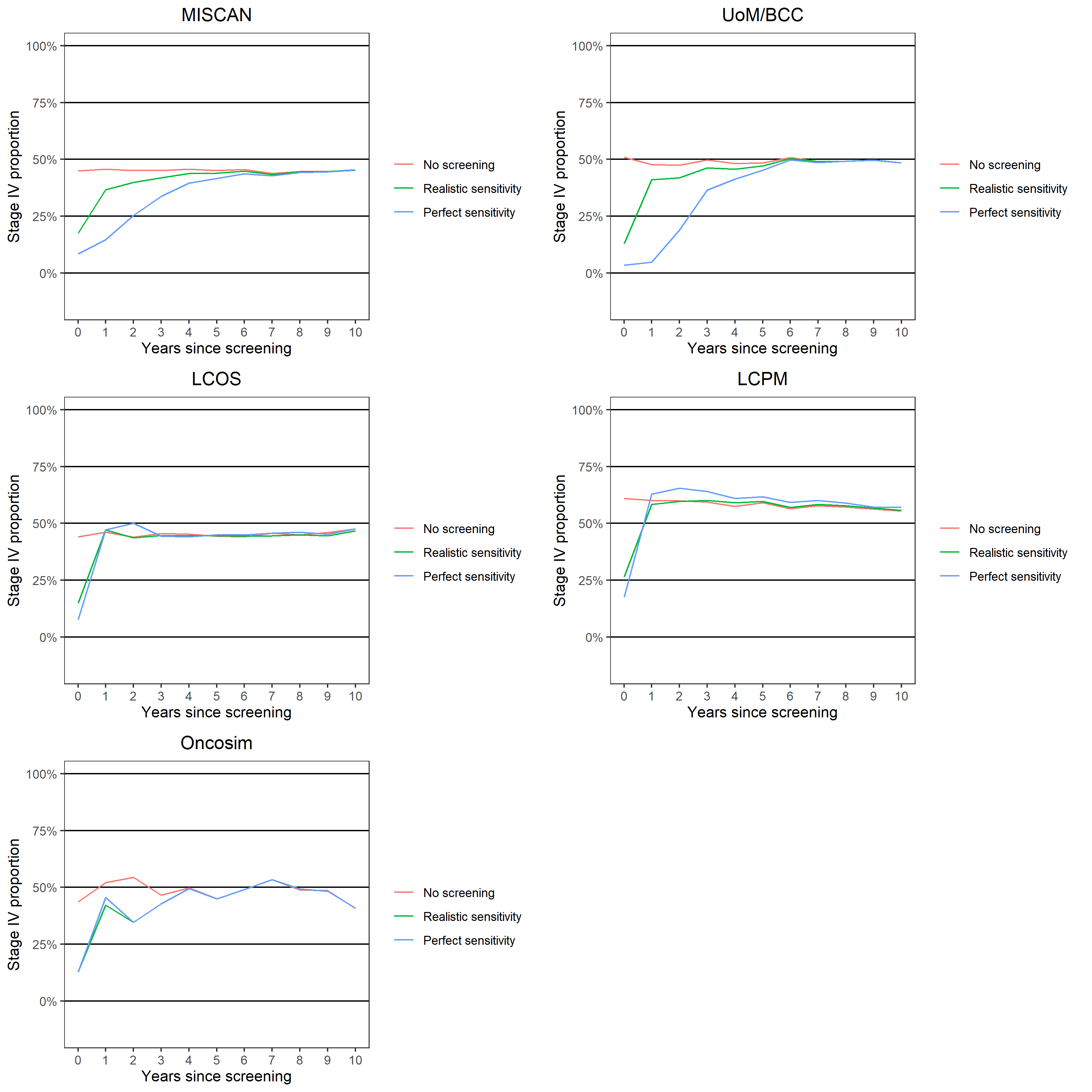

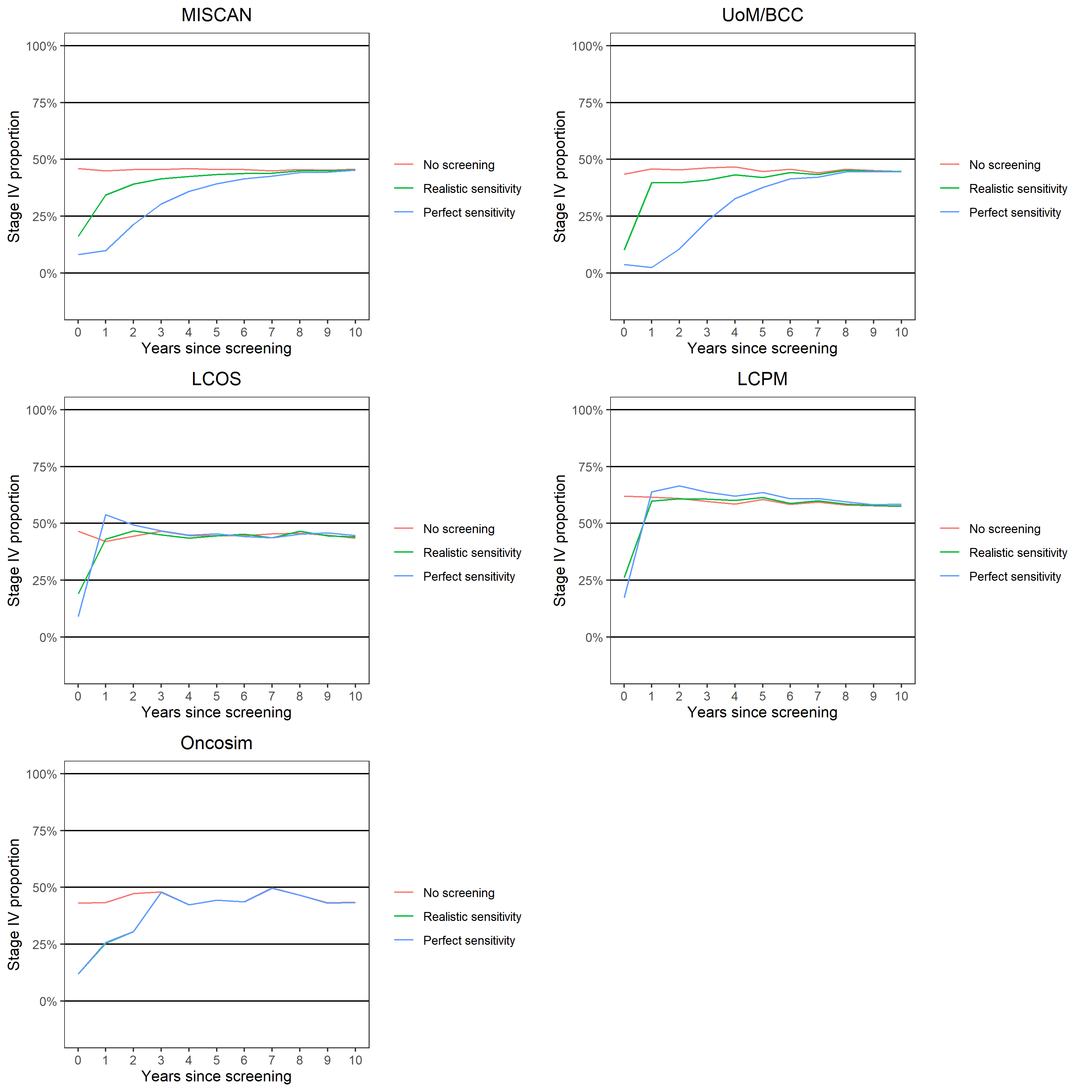
**

**Supplementary Figure 12: 15-year model-specific lung cancer mortality reductions under different assumptions (heavy smokers, men versus women)**

**Men Women**

**
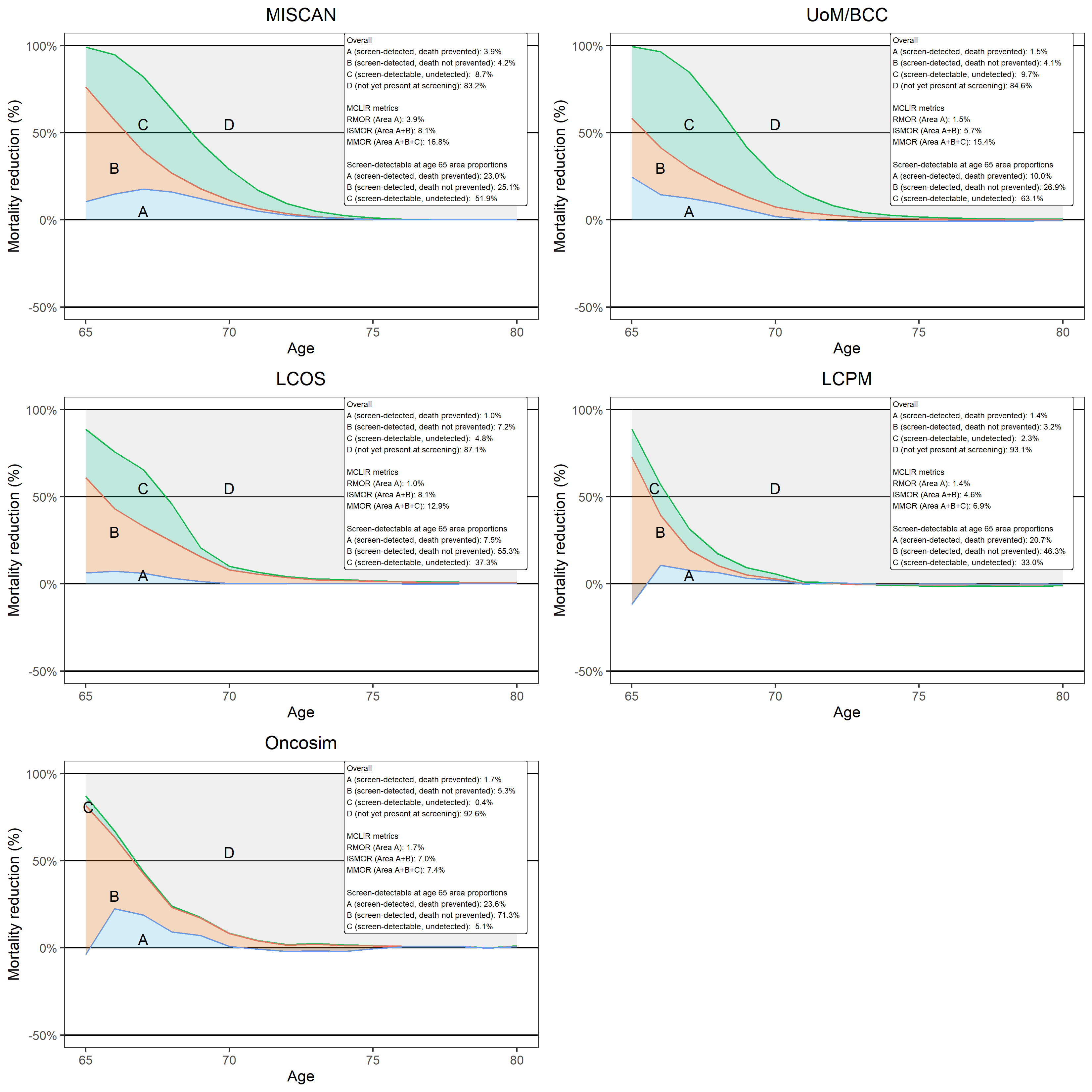

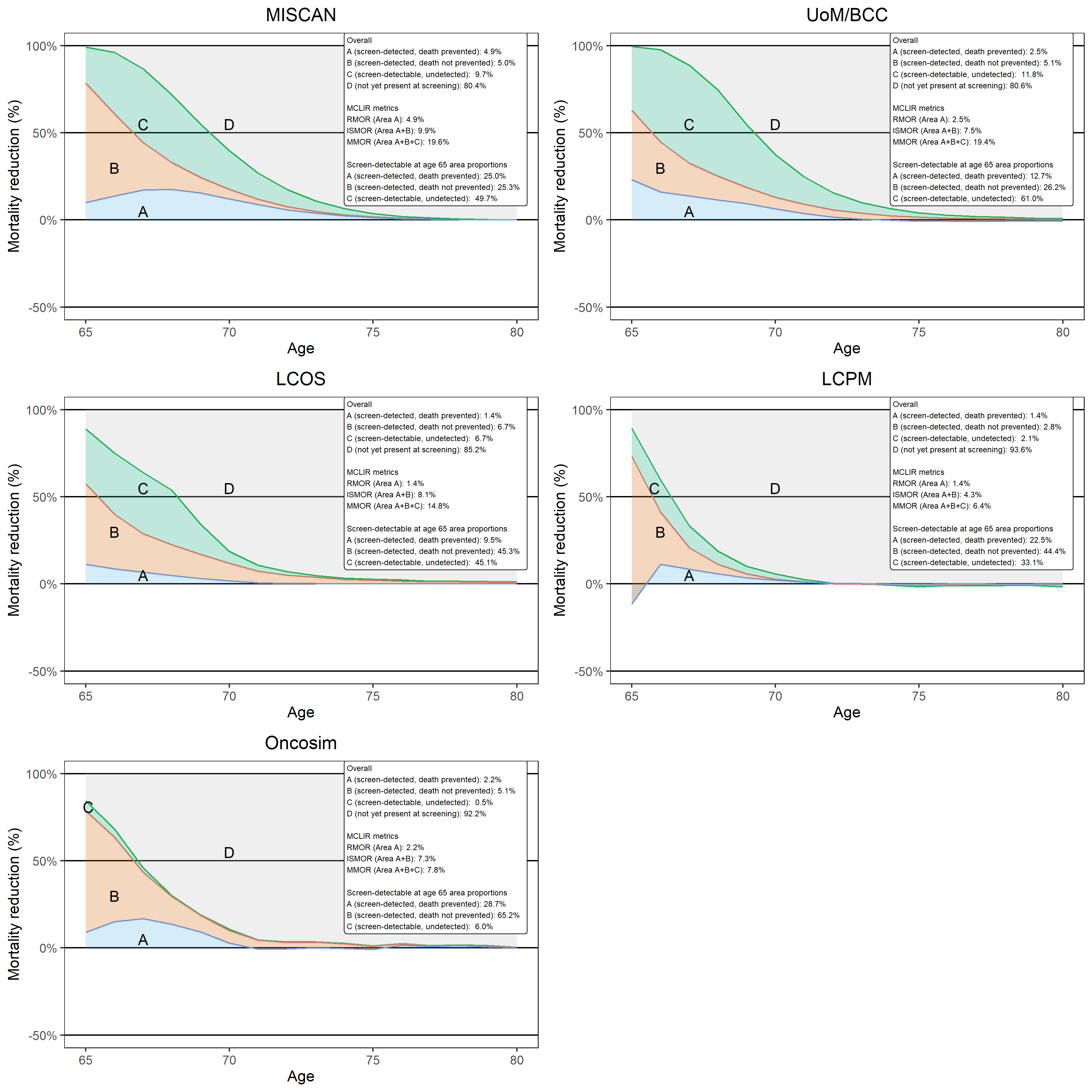
**

**Supplementary Figure 13: 15-year model-specific lung cancer mortality reductions under different assumptions (light smokers, men versus women)**

**Men Women**

**
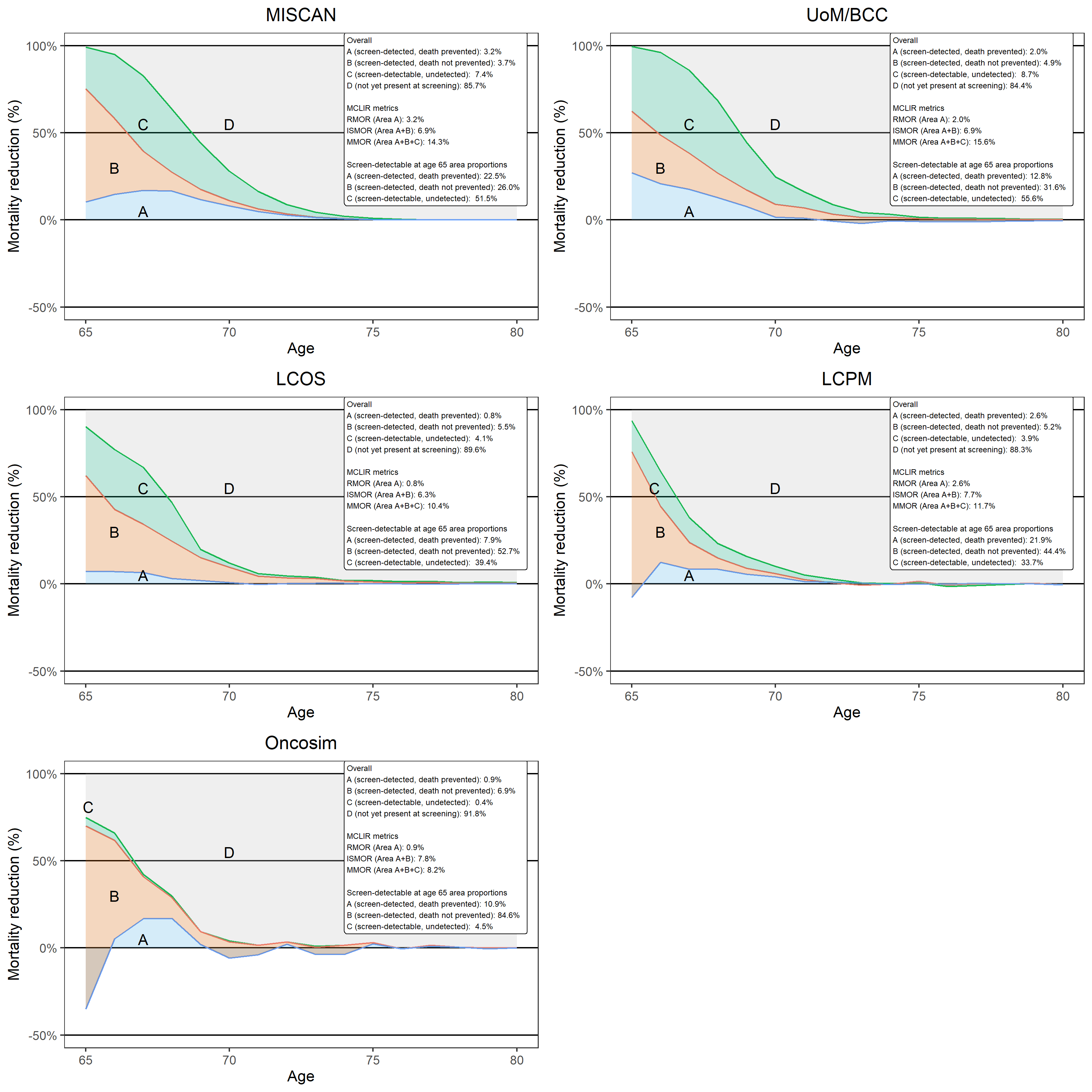

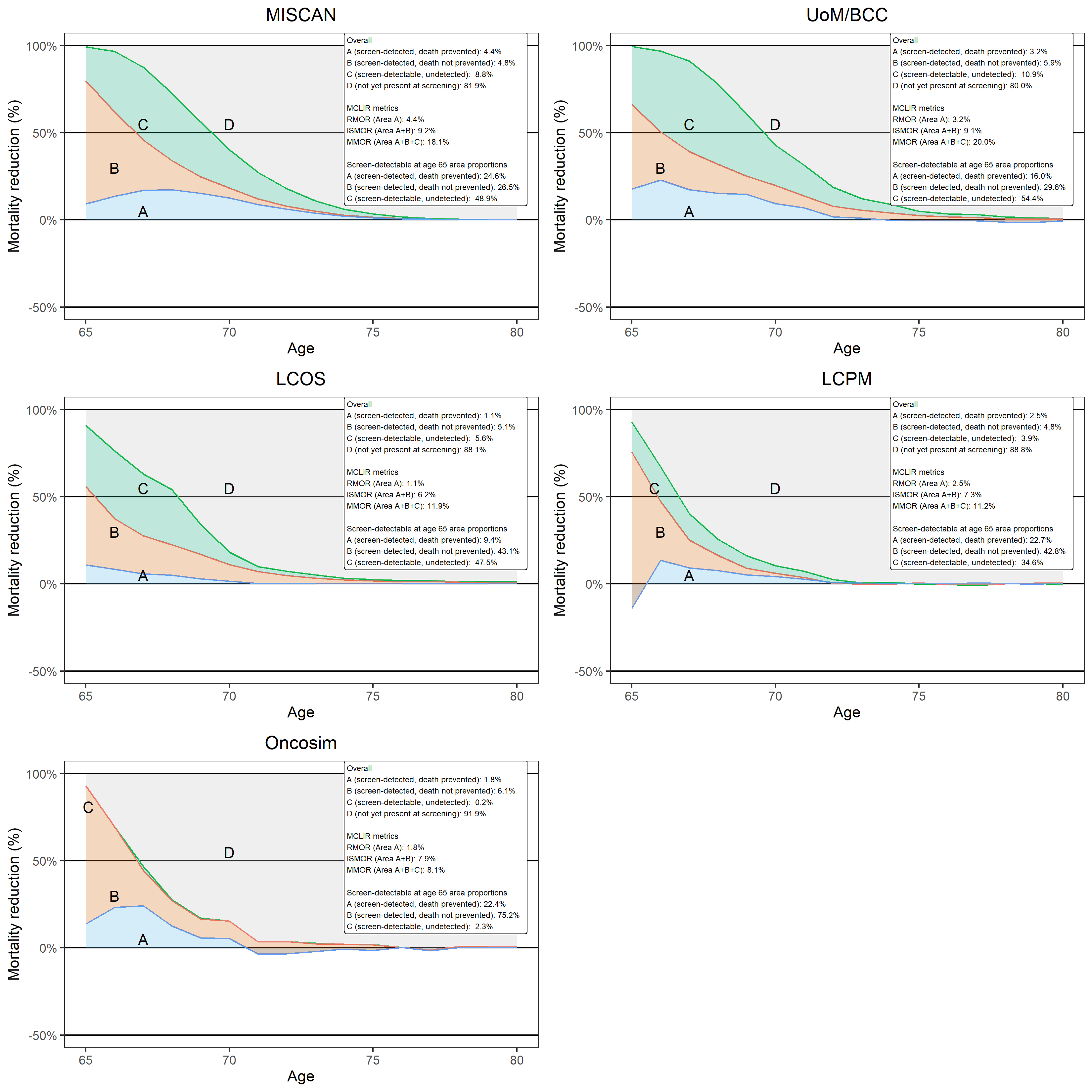
**

**Supplementary Figure 14: 15-year model-specific lung cancer incidence reductions under different assumptions**

**(heavy versus light smokers)**

**Heavy Light**

**
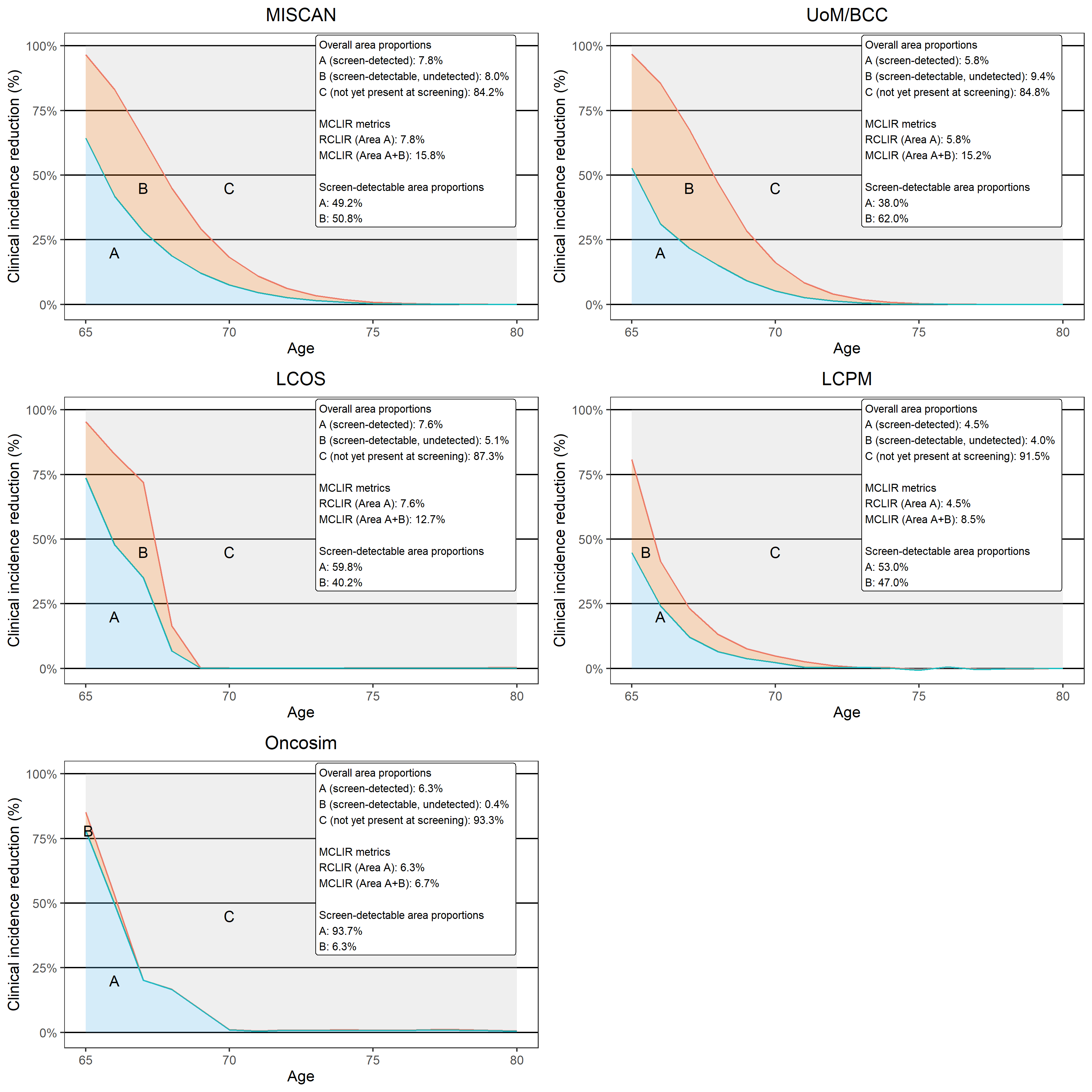

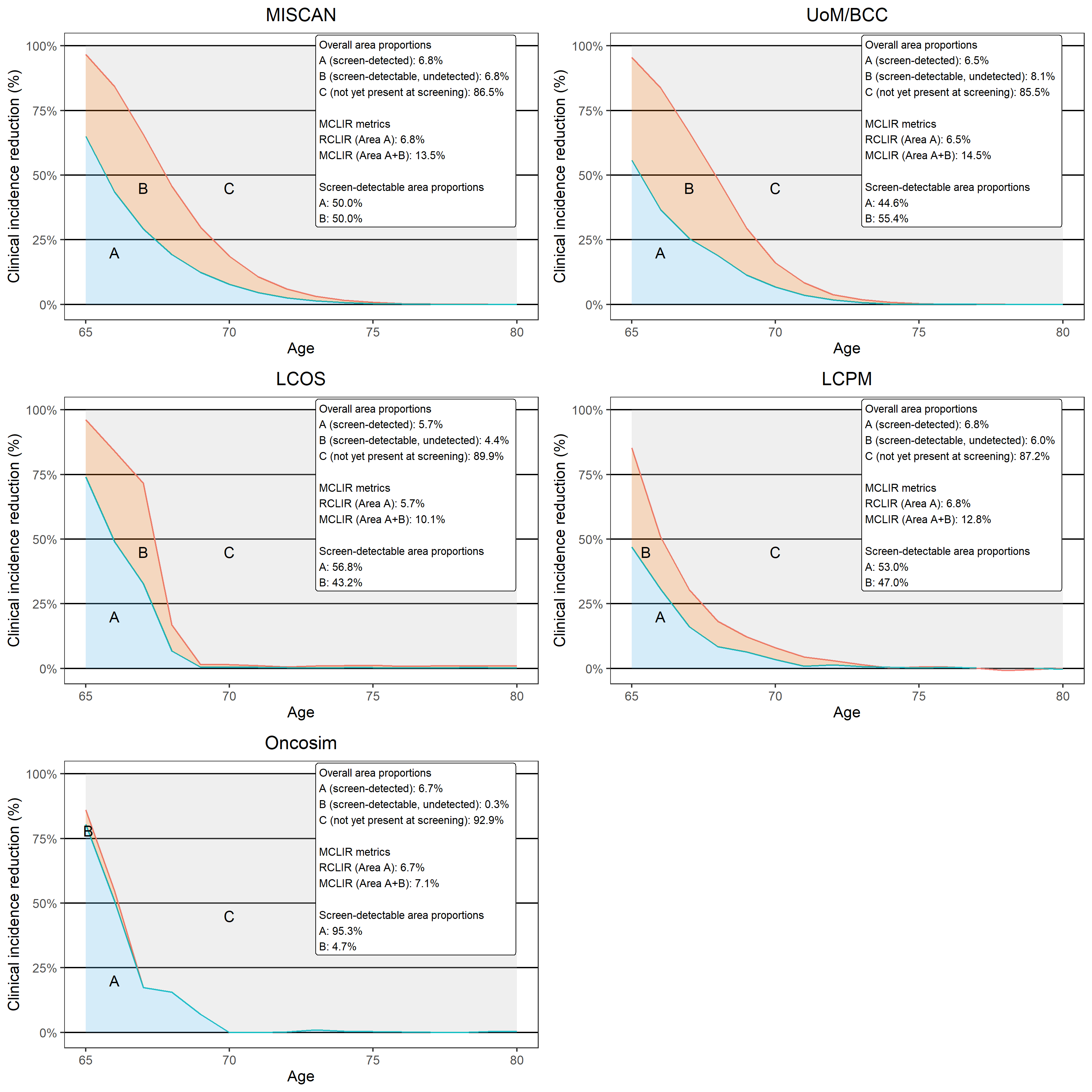
**

**Supplementary Figure 15: stage distributions in the absence of screening (heavy versus light smokers)**

**Heavy Light**


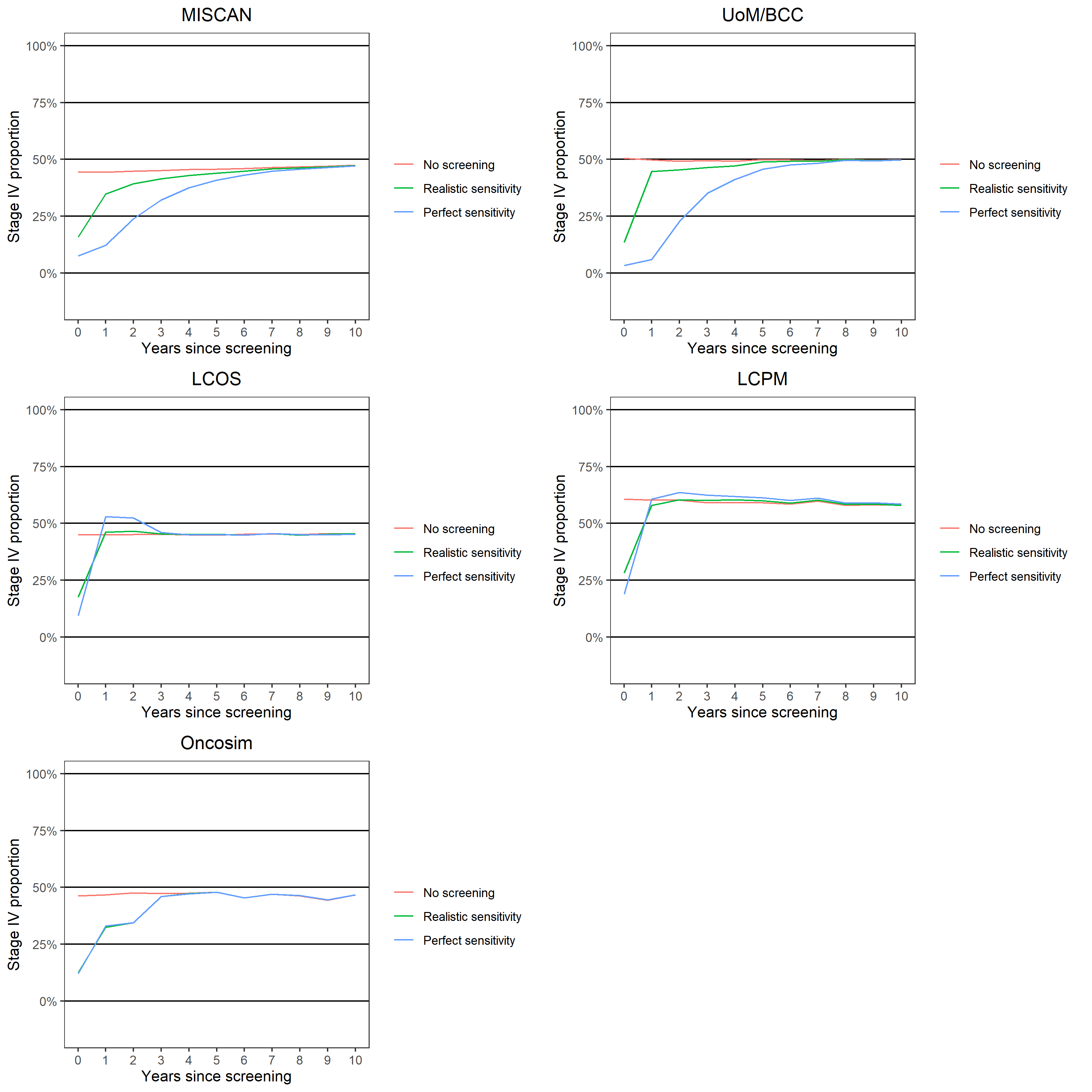

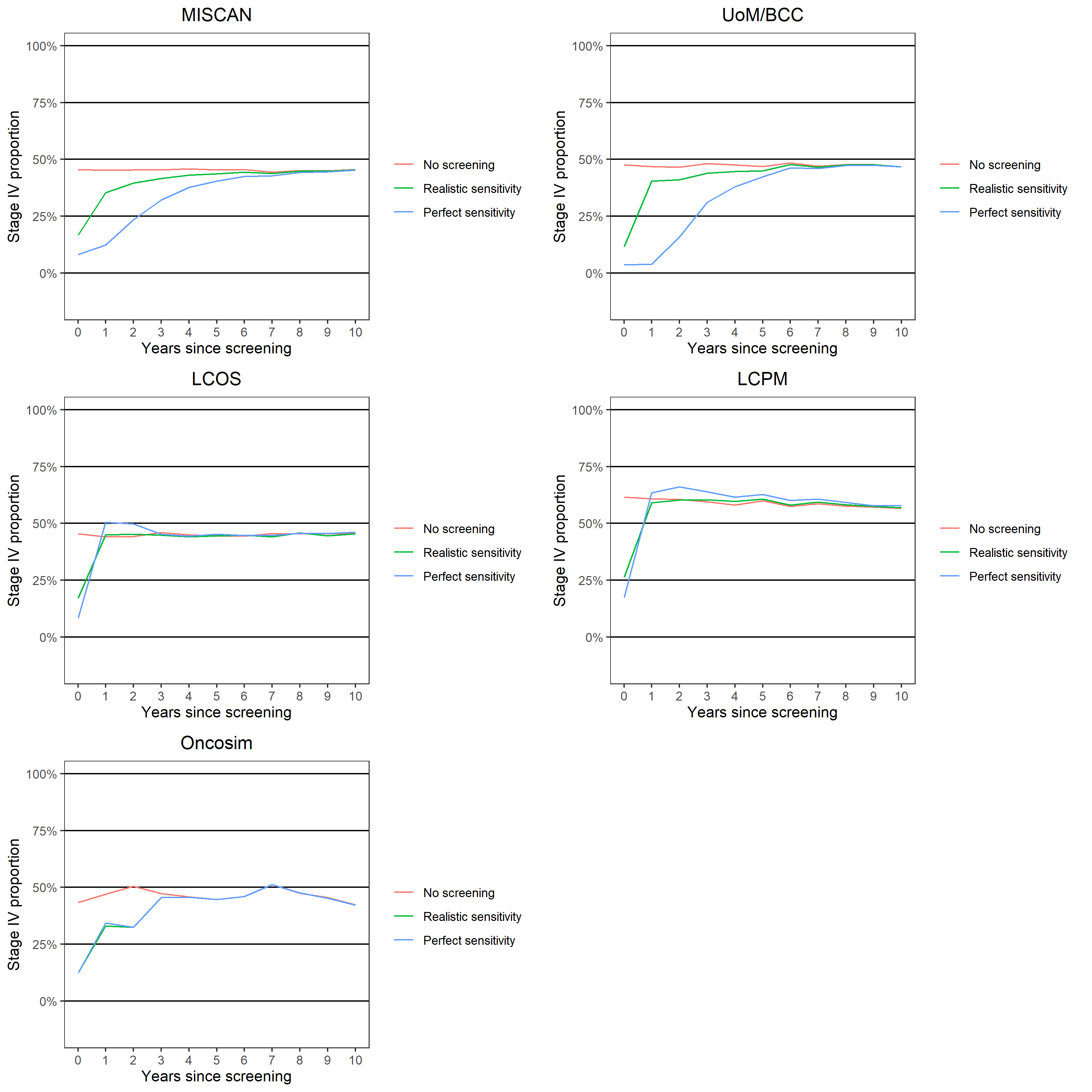


**Supplementary Figure 16: stage distributions of screen-detected cases under perfect sensitivity (heavy versus light smokers)**

**Heavy Light**

**
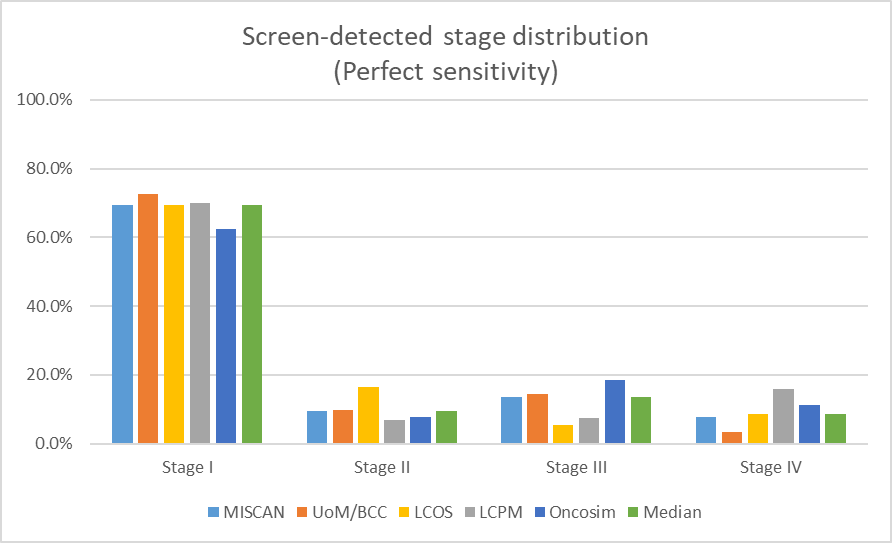

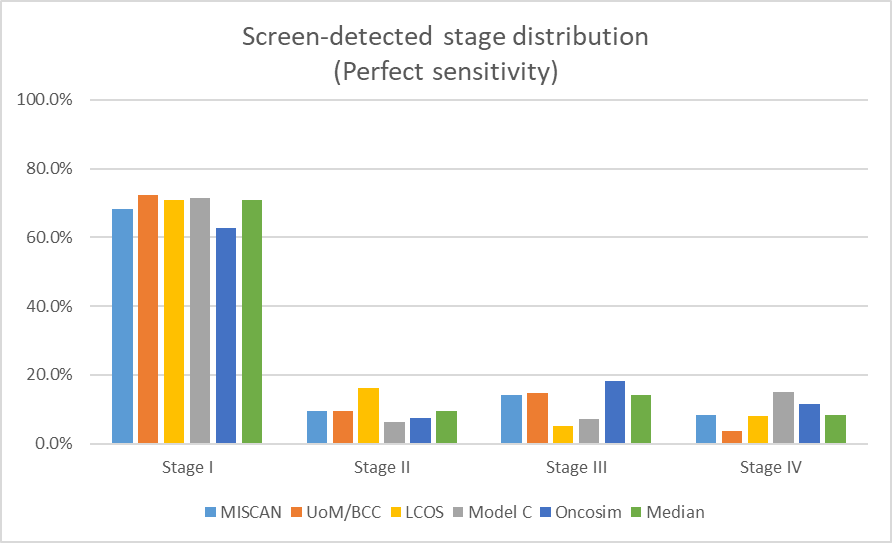
**

**Supplementary Figure 17: The effect of sensitivity on the occurrence of stage IV cancers post-screening (heavy versus light smokers)**

**Heavy Light**

**
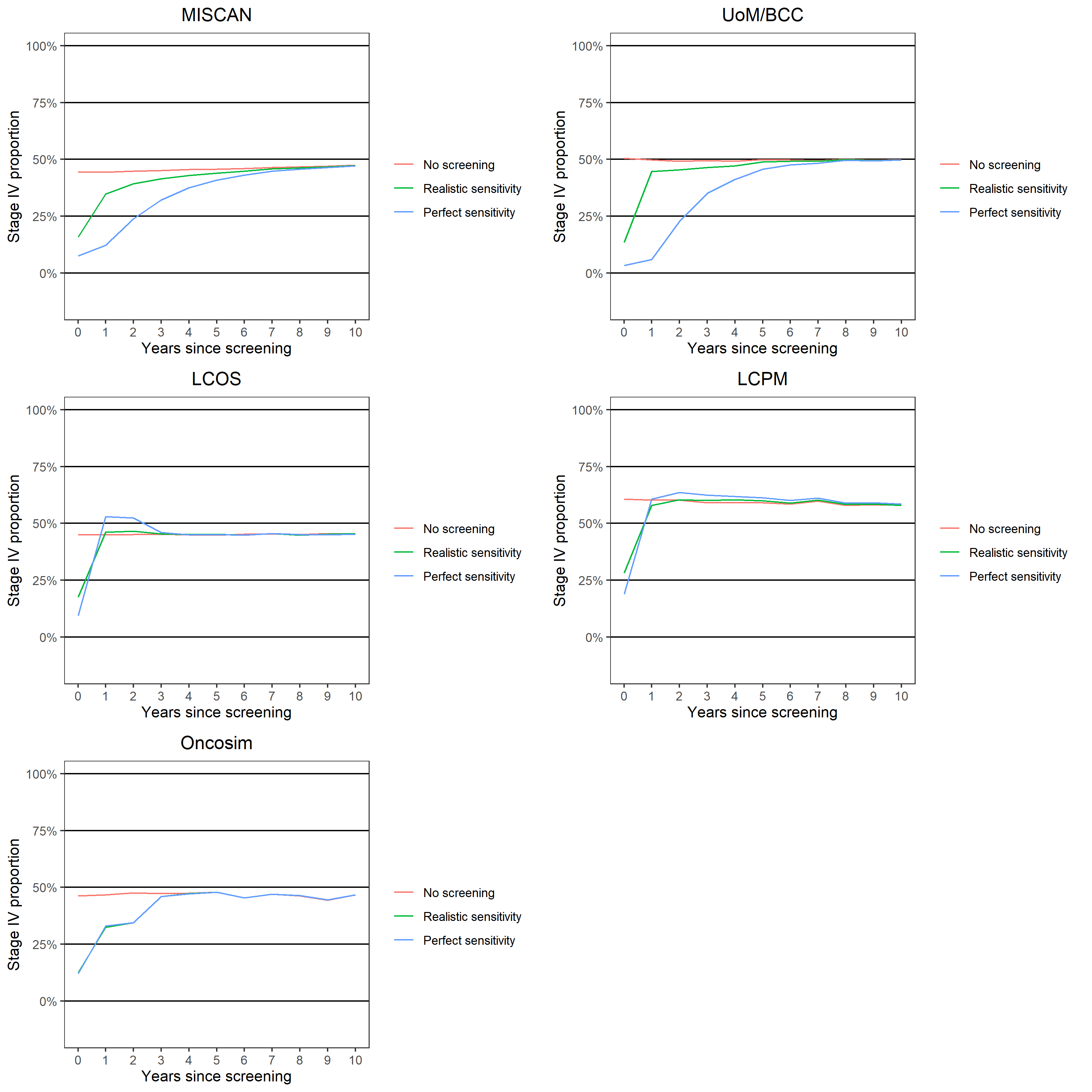

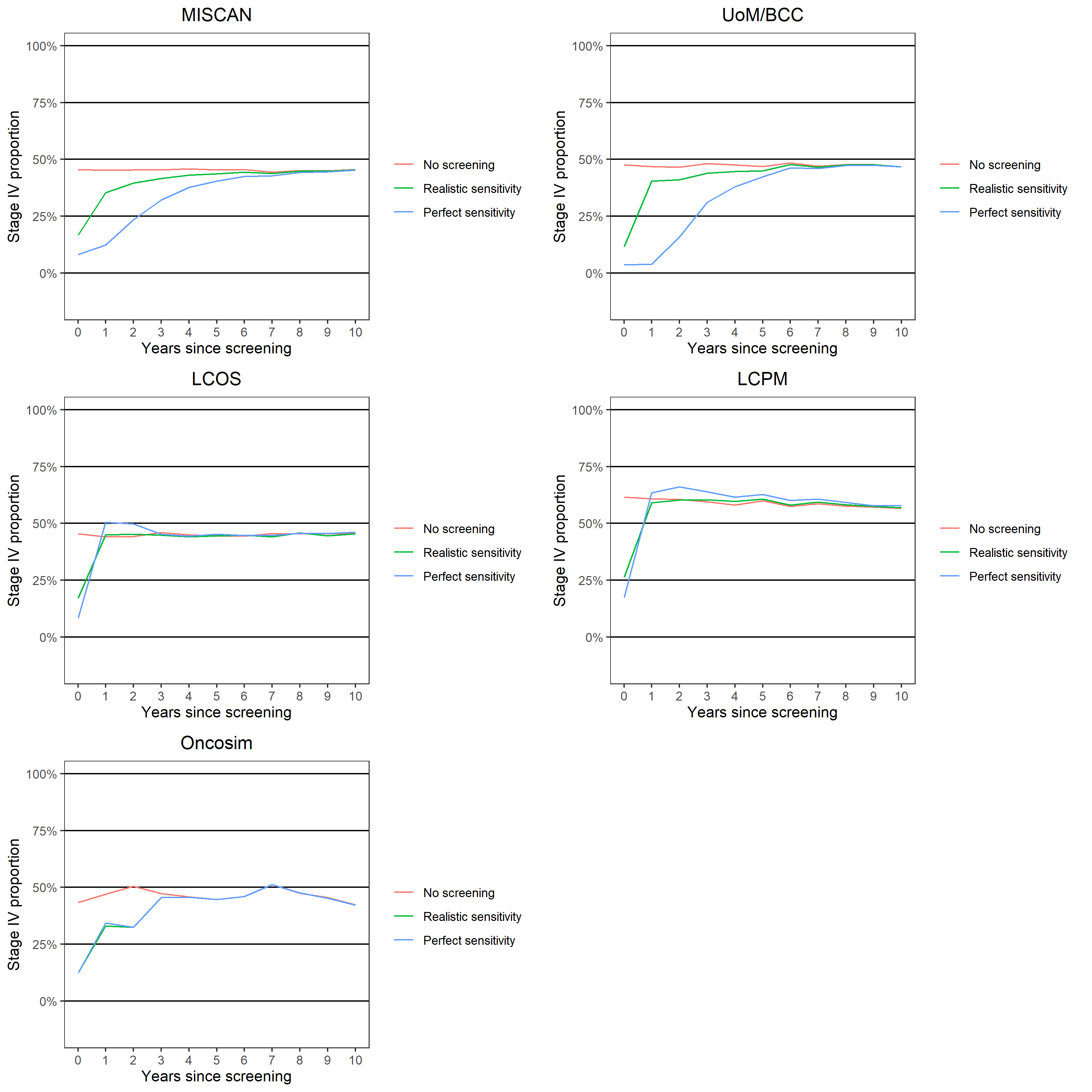
**

**Supplementary Figure 18: 15-year model-specific lung cancer mortality reductions under different assumptions (heavy versus light smokers)**

**Heavy Light**

**
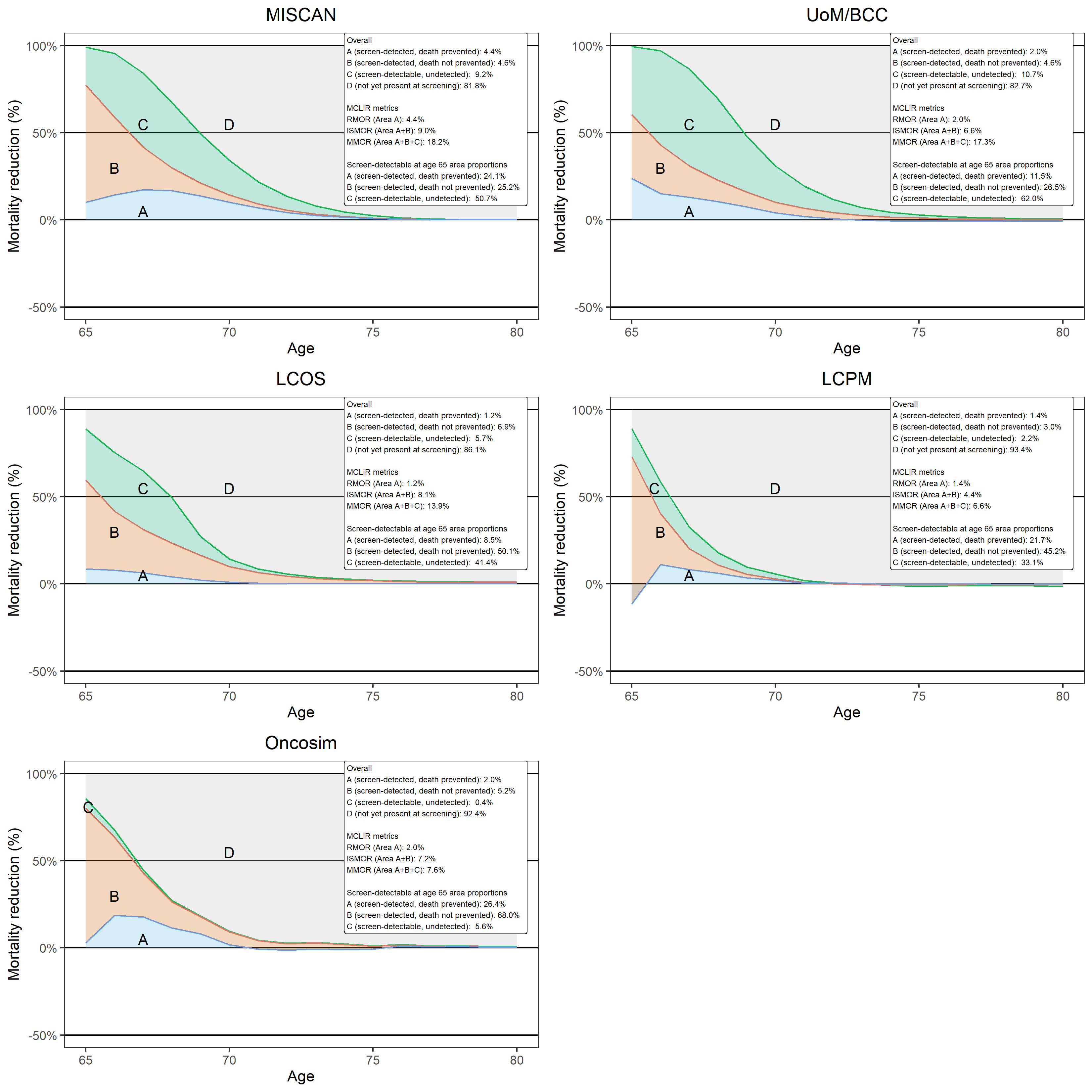

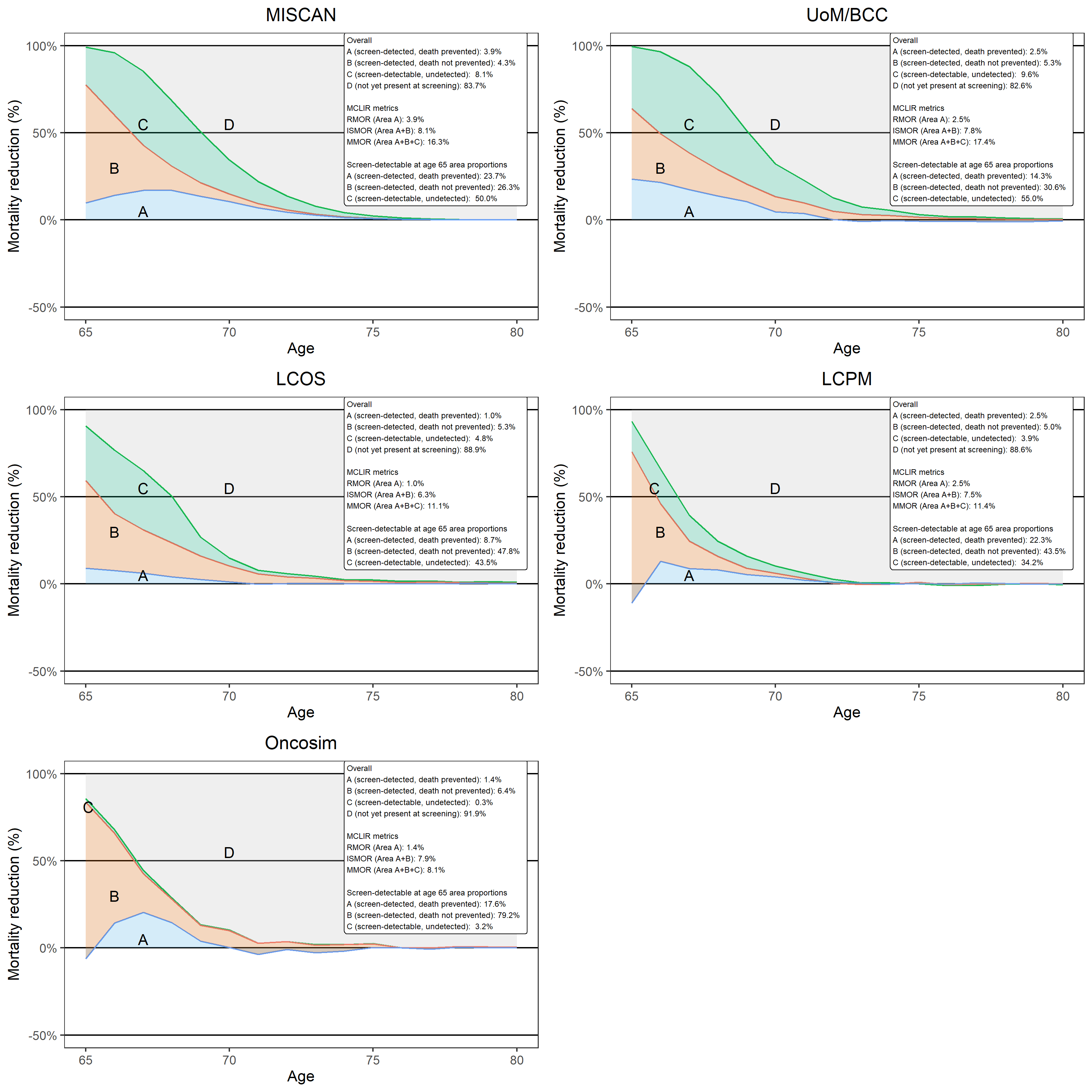
**
